# Temporal change in urban fish biodiversity—Gains, losses, and drivers of change

**DOI:** 10.1002/ece3.10845

**Published:** 2024-02-06

**Authors:** Lauren Lawson, Christopher B. Edge, Marie‐Josée Fortin, Donald A. Jackson

**Affiliations:** ^1^ Department of Ecology and Evolutionary Biology University of Toronto Toronto Ontario Canada; ^2^ Natural Resources Canada Canadian Forest Service Atlantic Forestry Center Fredericton New Brunswick Canada

**Keywords:** fish community ecology, freshwater ecology, temporal biodiversity, urban ecology, urban rivers, watersheds

## Abstract

Our aim was to examine temporal change in alpha and beta diversity of freshwater fish communities in rivers that have urbanized over the same period to understand the influence of changes in land use and river connectivity on community change. We used biological (2001–2018), land use (2000–2015), and connectivity data (1987–2017) from Toronto, Ontario, Canada. We used linear mixed effects models to determine the strength of upstream land use, connectivity, and their changes over time to explain temporal change in alpha and beta diversity indices. We examined beta diversity using the temporal beta diversity index (TBI) to assess site‐specific community change. The TBI was partitioned into gains and losses, and species‐specific changes in abundance were assessed using paired *t*‐tests. There were more gains than losses across the study sites as measured by TBI. We found little to no significant differences in species‐specific abundances at aggregated spatial scales (study region, watershed, stream order). We found different relationships between landscape and connectivity variables with the biodiversity indices tested; however, almost all estimated confidence intervals overlapped with zero and had low goodness‐of‐fit. More fish biodiversity gains than losses were found across the study region, as measured by TBI. We found TBI to be a useful indicator of change as it identifies key sites to further investigate. We found two high value TBI sites gained non‐native species, and one site shifted from a cool‐water to warm‐water species dominated community, both of which have management implications. Upstream catchment land use and connectivity had poor explanatory power for change in the measured biodiversity indices. Ultimately, such spatial–temporal datasets are invaluable and can reveal trends in biodiversity useful for environmental management when considering competing interests involved with urban sprawl in the ongoing “Decade on Restoration.”

## Graphical abstract

We assess urban fish community change over time and drivers of change related to urbanization including land cover change and connectivity. Using the TBI, species richness, Pielou's evenness, and Shannon diversity, we find that land cover and connectivity do not effectively explain community change. Overall, the TBI suggests there are more gains than losses in fish communities through time.

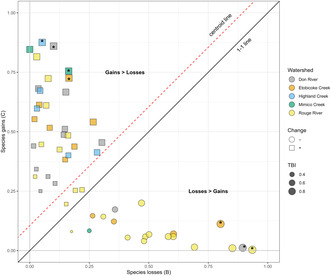

## 1 INTRODUCTION

Land‐use change is one of the leading causes of global biodiversity change (Jung et al., 2019). Urbanization is one of the most common and dramatic forms of land‐use change, and is characterized by increased human population density, increased artificial impermeable surface (e.g., roads, parking lots, and sidewalks), increased pollution, elevated temperature, and habitat fragmentation (Kondratyeva et al., 2020; Walsh et al., 2005). These changes can have profound impacts on all ecosystems experiencing urbanization. Land‐use change can be used as a proxy for numerous finer‐scale aspects of urban change as several environmental variables that drive ecological change are often correlated with land‐use change. For example, transitioning from forest to urban land cover is expected to impact hydrological dynamics and lead to more runoff, thus increasing the concentration of and types of contaminants flowing into water bodies (Arnold & Gibbons, 1996; Walsh et al., 2005). Potential ecological stressors include the concentration of the contaminant, and knowledge of both requires intensive sampling which is difficult to conduct at large spatial scales. Calculating land‐use change can be done consistently over large spatial extents through remote sensing and can reveal broader patterns of change across a watershed (Turner et al., 2007). The downside of using land‐use change as a proxy for ecological change is the potential loss of fine‐scale variation; however, the benefit of increased power to detect change and draw inferences over large spatial extents is appealing.

Currently, 56% of the global human population (83% in North America) lives in urban regions, and this is expected to grow to 68% by 2050 (89% in North America), making further urbanization imminent (Khor, 2022). Understanding how organisms respond to urban pressure is a fundamental ecological goal in the ongoing United Nations “Decade on Restoration” (2021–2030). This is particularly crucial for freshwater organisms, which are estimated to have declined by 84% since the 1970s (WWF, 2020). The interaction between further urbanization and restoration goals is important to consider, especially due to the increased valuation of urban green and blue spaces, which are naturalized vegetated spaces such as parks and forests (green), and aquatic spaces like ponds, wetlands, and rivers (blue). The value of urban blue space was recently recognized within the 2023 Kunming‐Montreal Global Biodiversity Framework (GBF), demonstrating the high level of global concern for the ecological integrity of urban water bodies (CBD, 2022). Target 12 of the GBF calls for more biodiversity‐inclusive planning and greater access to higher‐quality blue and green spaces in urban regions (CBD, 2022). Target 19 calls for more effective resource mobilization, including domestic resource mobilization for conservation, and Target 21 reiterates the importance of biodiversity monitoring (CBD, 2022). Regional specific biodiversity monitoring data provide opportunities to study how urbanization causes ecological changes, which in turn informs how resources can be effectively mobilized for conservation research and better biodiversity‐inclusive urban planning into the future.

We leveraged regional monitoring data to further the understanding of how freshwater ecological communities have changed in urban regions. We analyzed trends in alpha and beta diversity in a large urban region using long‐term fish community monitoring data from a local watershed management agency. Fish species distributions are closely tied to water quality and stream condition and thus change in fish communities can be indicative of broader environmental changes (Trautwein et al., 2012). Further, long‐term datasets collected through standardized sampling methodology provide invaluable opportunities to study ecological change (Hughes et al., 2017). The standardized methodology of long‐term monitoring datasets provides critical data which can be compared across time facilitating confident conclusions on biodiversity change (Turner et al., 2015). Long‐term ecological datasets allow for comparisons of within‐site change, instead of relying on space‐for‐time substitutions which are common in urban ecological studies when long‐term datasets are unavailable (Damgaard, 2019). Temporal datasets better control for aspects of site uniqueness, as many urban sites are impacted differently through time due to historic patterns of land‐use (i.e., legacy effects), which may lead to artifacts in space‐for‐time substitution approaches (Foster et al., 2003).

Using several measures of biodiversity change better encapsulates ecological change as each provides a different interpretation or estimate of the same underlying data patterns (Lyashevska & Farnsworth, 2012). Alpha diversity measures community diversity at a particular site, with different metrics placing emphasis on the number of species or the abundance of individual species (Jost, 2007). To capture community change, measures of alpha diversity can be compared among sites or at the same site through time. However, when alpha diversity is considered alone, information on species identity can be lost. Beta diversity measures community variation among sites (Koleff et al., 2003), or among time periods at the same site, with comparisons across space being more common, in part due to a lack of long‐term within‐site data. Importantly, measures of beta diversity can retain information on species identity, providing information on the loss and gain of individual species at sites (Baselga, 2008, 2010). Long‐term datasets with repeated measures at multiple sites allow for comparisons across both space and time (Hughes et al., 2017). Spatial–temporal datasets like the one we use are becoming more common as long‐term local monitoring datasets mature. Used in tandem, alpha and beta diversity indices are useful to understand broad patterns of biodiversity change through both number of species and composition of communities (Magurran, 2021).

Throughout history, urban centers have been tied to water (Hein, 2016). Living near freshwater provides access to drinking water, irrigation sources, transportation corridors, and cultural practices (Hein, 2016). Expansion of cities to accommodate increased human population growth, and rapid increases in migration from rural to urban regions in recent decades, has led to increased density of humans on the shores and in the watersheds of freshwater systems (Haidvogl, 2018). As a result, freshwater rivers and lakes have become degraded over time through multiple mechanisms including fragmentation due to small dams and mills, anthropogenic pollution (e.g., sewage, industrial waste, and household waste), and increased prevalence of introduced non‐native species (Haidvogl, 2018). The removal of soils and natural vegetation and increases in impermeable surfaces have fundamentally reduced, if not eliminated, the hydrological buffering capacity of urban watersheds leading to flashy freshwater systems prone to flooding (Arnold & Gibbons, 1996). Changes in hydrological dynamics, species dispersal ability due to in‐stream barriers, and increased pollutant loading can inevitably alter ecological dynamics in freshwater bodies, often causing declines in species sensitive to environmental change (Walsh et al., 2005).

Hydrological connectivity is important for riverine dynamics as changes in connectivity impact not only species dispersal patterns but also nutrient cycling and productivity (Ward & Stanford, 1983). Reduced ecological connectivity due to historic mills and dams, and contemporary roadway culverts, buried stream sections, and dams impacts a variety of ecological processes such as access to spawning grounds, access to prey, and ability to avoid predators (Choy et al., 2018; Edge et al., 2017). At the metacommunity level, decreased connectivity can reduce species colonization of sites from larger source populations which may supplement smaller populations across a watershed (Brown & Swan, 2010). Therefore, isolated sites may see higher rates of local extinction due to the lack of immigration from other sites (i.e., less dispersal).

To understand how freshwater biodiversity is changing in a highly urbanized region, we examined changes in riverine fish communities and potential drivers of change across 15 years in Toronto, Ontario, Canada's largest urban center. Toronto has experienced increased urban sprawl since the beginning of European settlements in the region in the mid‐18th century (Careless, 1984). Urbanization progressed steadily from the northern shore of Lake Ontario northward throughout the region's river valleys (Careless, 1984). Through time, the City buried rivers in underground pipes and dredged or filled wetlands to reduce exposure to polluted water and increase developable lands (Bonnell, 2014). Three large river systems and several smaller systems are found in Toronto, of which each has varying degrees of changes in their land‐use history due to uneven patterns of urban sprawl through time. In our study, we compared alpha and beta diversity of fish communities through time and examined drivers of change through analyzing land‐use change due to wetland loss, woodland loss, and anthropogenic land intensification over the study time period. Additionally, we examined the impact of connectivity through time by considering dendritic connectivity and change in connectivity as a potential driver of fish community change. The first objective of our study was to examine whether fish communities are experiencing more gains or losses in species or abundance‐per‐species through time (Question 1). We predicted fish communities would show more losses than gains due to increased urbanization in the study region over time and changing environmental quality ultimately contributing to species decline and local extirpation. Our second objective was to determine whether changes in upstream catchment land use and dendritic connectivity explained variation in fish community indices through time (Question 2). We predicted land‐use change, specifically loss of more natural land‐use cover (woodland, and wetland), would best explain variation in within‐site biodiversity through time.

## 2 METHODS

### 2.1 Fish community data sampling

**FIGURE 1:**
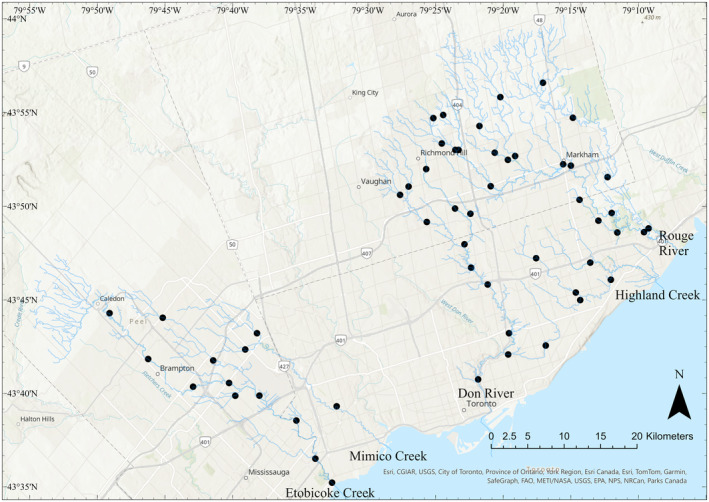
Map of study sites across Etobicoke Creek, Mimico Creek, Highland Creek, the Don River, and the Rouge River. Black points represent study sites. Blue lines represent river or creek watercourse.

Fish community data were obtained from the Toronto and Region Conservation Authority through their Regional Watershed Monitoring Program (TRCA, 2022). Sites in the same watershed were sampled once every 3 years, and watersheds were sampled in different years. Fish sampling was performed using the Ontario Stream Assessment Protocol and single‐pass electrofishing (Jarvie & Jackson, 2022; Stanfield, 2010). Fifty‐four sampling locations from five watersheds were included in our analysis: Etobicoke Creek (*n* = 11), East Don River (*n* = 13), Highland Creek (*n* = 5), Mimico Creek (*n* = 3), and the Rouge River (*n* = 22) (Figure 1). Individuals were identified to species, and abundances were tallied by the TRCA. Where samples were not identified to species level, species were assigned based on site history and/or expert knowledge or were removed from the dataset (see Table S1 for details). Fish data were summarized at the site level for two different time periods, T1: 2001–2003 (T1) and T2: 2016–2018 (T2). Each time period included one sampling event per site. Site Strahler order ranged from 1 to 6.

### 2.2 Ecological diversity indices

Species occurrence and abundance data were used to compute three alpha diversity metrics at T1 and T2: species richness, Pielou's evenness, and Shannon diversity. Richness measures the number of species occurring within a site. Pielou's evenness measures the relative abundances of species and is bounded between 0 and 1, with 1 indicating a completely even community (Jost, 2010). Pielou's evenness was calculated through the equation J = H′/log(S) where J is Pielou's evenness, S is richness, and H′ is the Shannon diversity index. The Shannon diversity index accounts for both species richness and relative abundance, and is considered to be more influenced by richness than Pielou's evenness in relatively small communities (Dejong, 1975). Each metric is commonly used to summarize biodiversity, and there are benefits and downsides to each (Magurran, 2012). Using all three metrics simultaneously better encapsulates the structure and change of biological communities. Alpha diversity metrics were calculated with the vegan package (Oksanen et al., 2020) using the specnumber() function for Pielou's evenness and diversity() for Shannon's diversity.

Change in fish community composition over time was determined using the temporal beta diversity index (TBI) (Legendre, 2019). To calculate the TBI, a relevant dissimilarity index is chosen, and a dissimilarity matrix is created. We use percent difference (i.e., Bray–Curtis dissimilarity), which has been shown to be well suited to analyze community data (Legendre, 2019). When using percent difference dissimilarity, the direction of community change within a site can be calculated through *D*%diff = (*B* + *C*)/(2*A* + *B* + *C*), where *D*%diff is percentage difference, *B* is where abundance is higher in T1 than T2 (losses), *C* is where abundance is higher in T2 than T1 (gains), and A is common abundance between T1 and T2 (similarity) (Legendre, 2019). Larger values of the TBI (*D*%diff) indicate more change between T1 and T2. Significance of change within a site between time periods in relation to other sites can be tested using a permutation approach as outlined by Legendre (2019). This approach uses a one‐tailed test of significance toward the upper tail; thus, only sites with exceptionally large change will be found to be “significantly” different (Legendre, 2019). Permutation is then followed by multiple test correction, wherein we used a Holm correction for multiple testing (Legendre, 2019). Exceptional sites can then be analyzed further to understand what underlying processes may be driving potential change, we consider both pre and post multiple testing correction results (see Legendre (2019) for details). The TBI was calculated using the TBI() function within the *adespatial* package of R using the method parameter set to percent difference (%difference) and 9999 permutations (Dray et al., 2021; Legendre, 2019).

### 2.3 Land cover

Land‐cover data were derived from the Southern Ontario Land Resource System, created by the Ontario Ministry of Natural Resources and Forestry. Land‐cover categories for T1 were derived from SOLRIS v1.2 by condensing definitions into three categories: anthropogenic, woodland, and wetland (Table S2). Land‐cover change between T1 and T2 was derived from SOLRIS v3.0 (OMNRF, 2019) by condensing the change categories into three categories: anthropogenic intensification, woodland loss, and wetland loss (Table S3). Both datasets are to a resolution of 0.5 hectares (OMNRF, 2019). Percentage land‐cover change per land‐use category was measured as the change in land cover per category divided by the watershed area per site. Anthropogenic intensification represents anthropogenic lands which were further urbanized, for example, if an area changed from permeable urban to impermeable urban. Land‐cover data were summarized at the entire upstream catchment level for each site through ArcGIS hydrotools (Figures S1 and S2). ArcGIS Pro Version 3.0.3 was used for all land cover analyses.

### 2.4 Connectivity

Dendritic connectivity was estimated using the Dendritic Connectivity Index (DCI) (Cote et al., 2009). For each stream segment, we calculated DCIs, a measure of how well an individual stream segment is connected to all other stream segments in 1987 (T1) (Choy et al., 2018) and 2017 (T2) (Edge et al., 2017). The DCI at T2 was calculated by recording the location and measuring downstream pool depth and barrier height of all existing barriers. The DCIs at T1 were calculated by compiling a list of mitigated or removed barriers in the streams and adding those barriers to the surveyed list at T2. Mitigated or removed barriers were identified by contacting groups and agencies (e.g., Trout Unlimited, Ontario Streams, Ontario Ministry of Natural Resources, and Department of Fisheries and Oceans) that have been involved with barrier mitigation projects in the Toronto Region, reviewing permit applications under Ontario Regulation 166/06 in the Fisheries and Oceans Canada database, and reviewing the corporate records database of the Toronto and Region Conservation Authority. Additional details of the methodology to identify removed or mitigated barriers can be found in Choy et al. (2018). The permeability of each barrier was estimated using outlet drop and baseflow of the stream (Choy et al., 2018; Edge et al., 2017).

### 2.5 Statistical analyses

#### 2.5.1 Explanatory modeling

Linear mixed effects models were used to determine which variables best explained the change in alpha and beta diversity indices between T1 and T2. A Gaussian distribution was used for all models as the response variables, which are measures of change, were normally distributed. Candidate models included predictor variables of land‐cover categories at T1, land‐cover change categories between T1 and T2, dendritic connectivity at T1, and dendritic connectivity change between T1 and T2 (Table 1). The TBI compares two time periods; therefore the TBI itself is used as a temporal response variable (Legendre, 2019). Watershed was retained as a random effect for each response variable. Differences in diversity patterns between watersheds are possible due to varying spatial patterns of urbanization and restoration through time creating legacy effects (Foster et al., 2003).

**TABLE 1:** Candidate models included in model selection for each biodiversity metric.

Model name	Model variables
Global model	Anthropogenic.T1 + %Δ Anthropogenic + Wetland.T1 + %Δ Wetland + Woodland.T1 + %Δ Woodland + DCI.T1 + ΔDCI
Land‐cover model	Anthropogenic.T1 + %Δ Anthropogenic + Wetland.T1 + %Δ Wetland + Woodland.T1 + %Δ Woodland
DCI model	DCI.T1 + ΔDCI
Anthropogenic full model	Anthropogenic.T1 + %Δ Anthropogenic
Wetland full model	Wetland.T1 + %Δ Wetland
Woodland full model	Woodland.T1 + %Δ Woodland

*Note*: Watershed was included as a random effect in all models.

The candidate models were compared using an AIC model‐selection framework with a correction for small sample size (AICc) (Harrison et al., 2018; Symonds & Moussalli, 2011). Models were considered top models in our model‐selection framework if their ΔAIC < 2 (Burnham et al., 2011; Harrison et al., 2018). We tested models containing interactions between each variable and its change component; however, models with interactions had high variance inflation factors so the interactions were not included in the final candidate models (Table 1). All predictor variables were standardized to z‐scores prior to analyses. Model averaging was performed for all models with ΔAIC < 2 for each response variable, and subset estimates are reported. Model selection through AICc was performed using the function aictab() through the package AICcmodavg (Mazerolle, 2020) in R with models with ΔAIC < 2 retained for model averaging. Model averaging was performed using the function model.avg() from the package MuMIn (Barton, 2022; Harrison et al., 2018). *R* ^2^ for mixed models were calculated using the function r.squaredGLMM from the MuMIn R package (Barton, 2022; Harrison et al., 2018). R version 4.2.2 was used for all statistical analyses and visualizations.

#### 2.5.2 Individual species abundance

Changes in individual species abundance across the study region, each watershed, and each stream order were tested individually using paired *t*‐tests with Holm corrections for multiple testing (Holm, 1979; Legendre & Condit, 2019). This approach allows for the testing of trends in significant change in abundance of individual species through aggregating sites to a higher level of organization (e.g., study, watershed, and stream order). Paired *t*‐tests to analyze individual species change were computed with 9999 random permutations followed by a Holm correction for multiple testing through the *adespatial* package and functions paired.kranttest.Rpairedt.test.spec() and p.adjust() from the package stats (R Core Team, 2022).

## 3 RESULTS

### 3.1 Data summary

A total of 42 fish species were observed within the compilation of sites and time periods; the range in species richness observed differed among the sites within each watershed: Rouge River (2–17), Don River (2–8), Highland Creek (2–5), Mimico Creek (2–3), and Etobicoke Creek (2–13).

Changes in site species richness between the two time periods ranged between −7 and 6, (i.e., sites lost between 0 and 7 species, and gained between 0 and 6 species; Figure S3). Changes in Pielou's evenness between T1 and T2 ranged between −0.76 and 0.55 (Figure S3). Changes in Shannon diversity ranged between −0.76 and 1.57 (Figure S3). Across all sites, the TBI ranged between 0.26 and 0.96 (Figure S3).

### 3.2 Temporal beta diversity index

**FIGURE 2:**
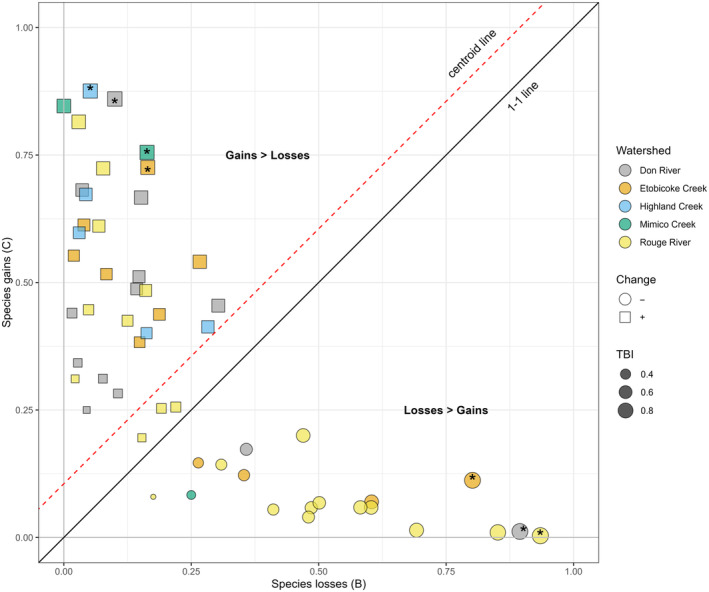
B‐C plot for the temporal beta diversity index (TBI). Squares represent sites where gains > losses. Circles represent sites where gains < losses. Colors denote watersheds. The location of the centroid line is above the 1–1 line, suggesting the system reports more gains overall than losses. Asterisks represent high TBI sites (prior to holm correction *p* < .05 sites). Size of points represents relative TBI values.

**FIGURE 3:**
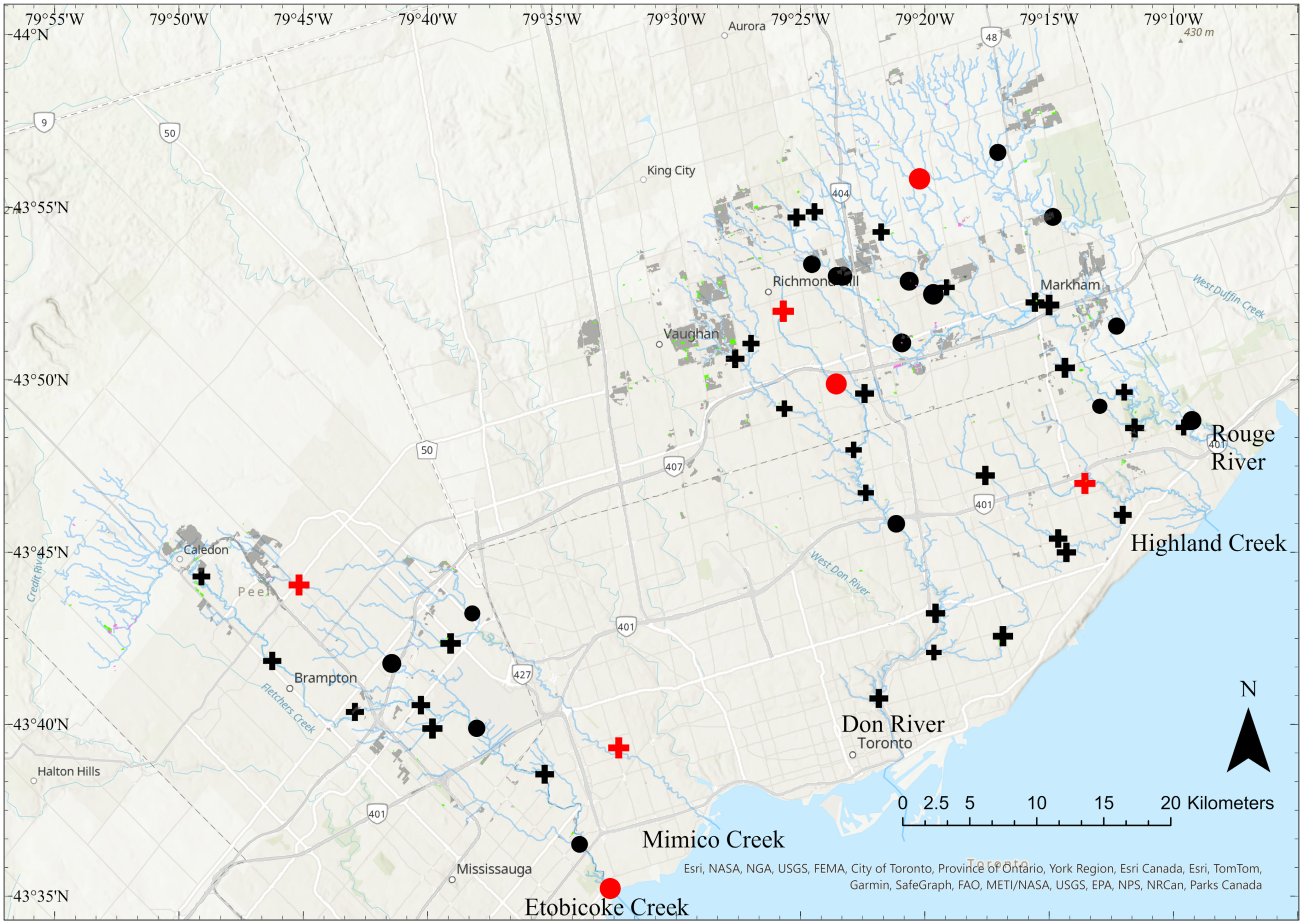
Map of sites with the temporal beta diversity index (TBI) results. Size represents relative TBI value. Plus sign sites have more gains than losses; circle points represent more losses than gains. Red sites represent high TBI sites (pre‐Holm correction significant sites). Gray polygons represent urban intensification. Green polygons represent woodland loss. Purple polygons represent wetland loss. Site identification numbers increase in the upstream direction.

Across all sites sampled, there were more gains than losses for species or abundances‐per‐species on average. Averaged gains (0.59) were larger than losses (0.41) (i.e., species gains averaged 59% and losses averaged 41%; Figure 2). We found seven sites with significant change in species or abundances‐per‐species (*p* < .05) prior to Holm correction (Figure 2). These included two sites on the Don River (DN011WM, DN010WMb), one site on the Rouge River (RG019WM), one site on Highland Creek (HL005WM), one site on Mimico Creek (MM002WM), and two sites on Etobicoke Creek (EC001WM, EC010WM) (Figure 3; Table S4). When the data were re‐analyzed using occurrence rather than abundance data, two sites were found to have significant change with one gain site (EC010WM) and one loss site (EC001WM) prior to Holm correction (Table S5). We present *p*‐values both pre‐ and post‐Holm correction for multiple testing, as both values may be of interest to ecologists. The TBI values themselves can be used in modeling without significance testing (see Kuczynski et al., 2018; Robichaud & Rooney, 2022); however, we chose to include significance testing for consideration. The 7 exceptional sites pre‐correction are subsequently referred to as high TBI sites, as the pre‐correction significant sites represent the seven highest TBI sites, all reporting a TBI value >0.89. These seven sites represent the sites with the largest compositional differences through time (Legendre & Condit, 2019).

### 3.3 Individual species change

Paired *t*‐tests were performed across all sites, within each watershed, and within each stream order with a correction for multiple testing. When corrected for multiple testing, there were no significant differences in species abundances between the two time periods when sites were examined at the study level, watershed level, or stream order level (Tables S6–S8) other than a slight mean decrease in white sucker (*Catostomus commersonii*) in the Don River watershed (Table S7).

For the high TBI sites, four had more gains than losses, and three had more losses than gains (Figures 2 and 3). There were no significant differences in species abundances between the two time periods when sites were compared across all high TBI sites (Table S9), to all high TBI sites with overall gains (Table S10), or all high TBI sites with overall losses (Table S11). A closer examination of species‐specific abundance changes across all the sampling sites revealed an increase in the invasive round goby (*Neogobius melanostomus*) from 0 to 78 with 74 sampled at one site (DN001WM), and an increase from 0 to 4 sampled at one high TBI site (EC001WM). Additionally, introduced green sunfish (*Lepomis cyanellus*) increased in two high TBI sites (EC001WM and EC010WM) to abundances of 1 and 255, respectively.

### 3.4 Model results

**FIGURE 4:**
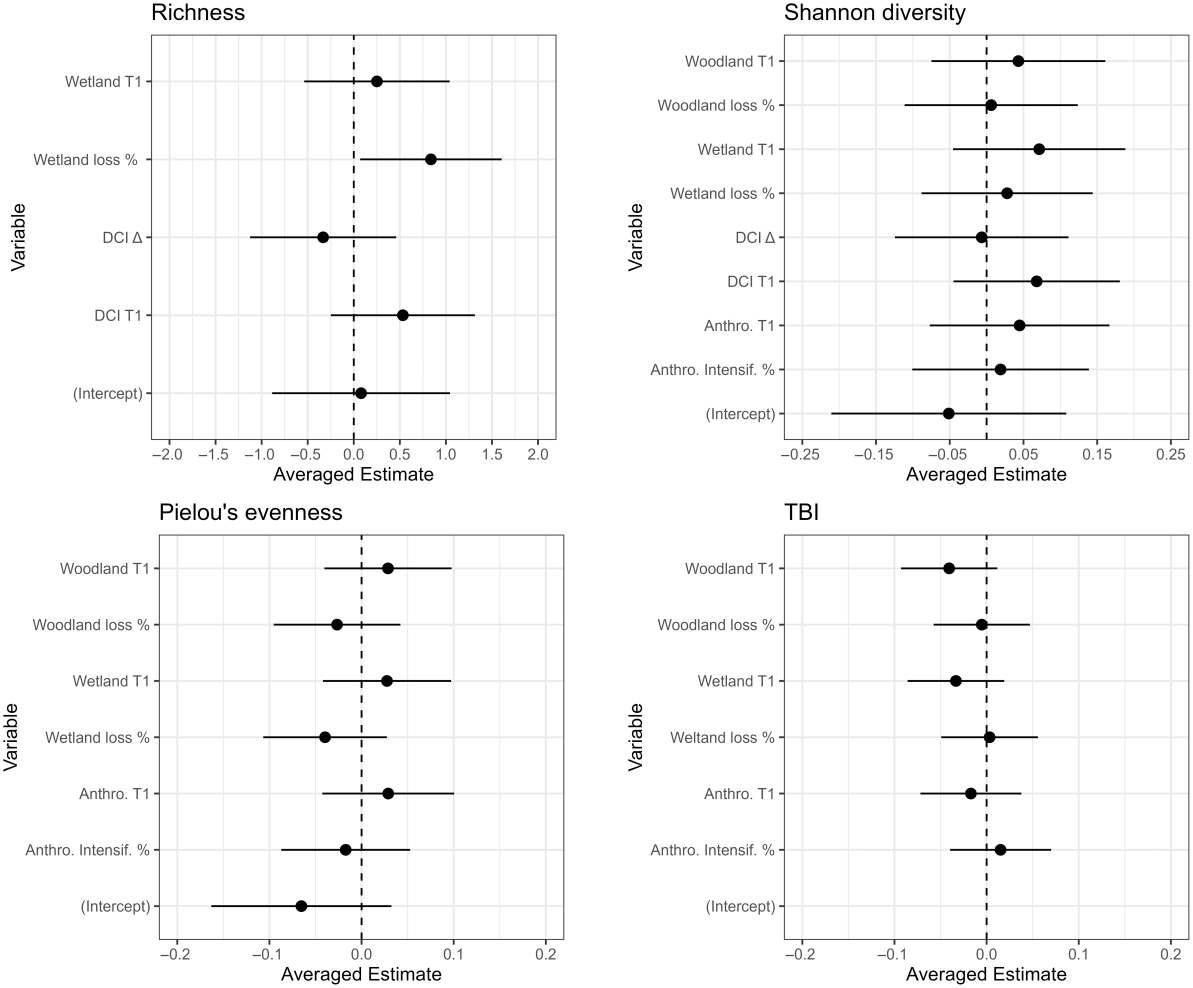
Model‐averaged estimates for richness, Shannon diversity, Pielou's evenness, and temporal beta diversity index (TBI) with 95% confidence intervals. The TBI intercept value of 0.62 with 95% CI [0.57, 0.67] not shown due to data visualization limitations. All estimates represent subset estimates.

The two averaged top models (Table 2) for richness indicated a positive relationship between change in richness and wetland at T1, wetland loss, and DCI at T1, although estimate 95% confidence intervals overlapped with zero except for wetland loss (Figure 4). There was a negative relationship between change in richness and DCI change, although the estimate 95% confidence intervals overlapped with zero (Figure 4).

**TABLE 2:** Model selection results for richness, Shannon diversity, Pielou's evenness, and the temporal beta diversity index (TBI).

Response	Model	*K*	AIC_c_	Delta_AICc_	AIC_cWt_	Cum.Wt	LL	$R_{m}^{2}$	$R_{c}^{2}$
Richness	Wetland	5	272.45	0.00	0.61	0.61	−130.60	.08	.13
	DCI	5	274.36	1.90	0.24	0.85	−131.55	.05	.07
	Woodland	5	276.55	4.09	0.08	0.93	−132.65		
	Anthro.	5	276.81	4.35	0.07	1.00	−132.78		
	Land cover	9	282.86	10.41	0.00	1.00	−130.39		
	Global	11	286.33	13.88	0.00	1.00	−129.02		
Shannon diversity	Wetland	5	67.30	0.00	0.33	0.33	−28.02	.03	.04
	DCI	5	67.58	0.28	0.28	0.61	−28.17	.02	.13
	Anthro.	5	68.28	0.98	0.20	0.81	−28.52	.01	.07
	Woodland	5	68.36	1.06	0.19	1.00	−28.55	.01	.09
	Land cover	9	77.73	10.43	0.00	1.00	−27.82		
	Global	11	82.58	15.29	0.00	1.00	−27.15		
Pielou's evenness	Wetland	5	9.72	0.00	0.39	0.39	0.76	.04	.11
	Woodland	5	10.55	0.83	0.26	0.65	0.35	.03	.10
	Anthro.	5	11.00	1.28	0.21	0.86	0.13	.02	.12
	DCI	5	11.77	2.05	0.14	1.00	−0.26		
	Land cover	9	20.02	10.30	0.00	1.00	1.04		
	Global	11	25.46	15.74	0.00	1.00	1.41		
TBI	Woodland	5	−19.44	0.00	0.40	0.40	15.34	.04	.04
	Wetland	5	−18.68	0.76	0.28	0.68	14.97	.03	.03
	Anthro.	5	−17.94	1.50	0.19	0.87	14.60	.02	.02
	DCI	5	−17.17	2.27	0.13	1.00	14.21		
	Land cover	9	−10.46	8.98	0.00	1.00	16.27		
	Global	11	−4.43	15.01	0.00	1.00	16.36		

*Note*: Anthro. represents anthropogenic model. Models with <2 delta AIC include *R* ^2^ values. Models <2 delta AIC were included in model averaging and data visualization.

The four averaged top models (Table 2) for Shannon diversity indicated positive relationship between change in Shannon diversity and woodland T1, wetland T1, wetland loss, woodland loss, anthropogenic T1, anthropogenic intensification, and DCI T1, although estimate 95% confidence intervals overlapped with zero (Figure 4). There was a slight negative relationship between change in Shannon diversity and DCI change, although estimate 95% confidence intervals overlapped with 0 (Figure 4).

The three averaged top models for Pielou's evenness indicate a positive relationship between change in Pielou's evenness and woodland T1, wetland T1, anthropogenic T1, although estimate confidence intervals overlapped with zero (Figure 4). There was a negative relationship with woodland loss, wetland loss, and anthropogenic intensification, although all confidence intervals overlapped with zero (Figure 4).

The three averaged top models (Table 2) for TBI indicate a negative relationship between TBI and woodland T1, woodland loss, wetland T1, and anthropogenic T1, although estimate confidence intervals overlapped with zero (Figure 4). There was a positive relationship between TBI and wetland loss and anthropogenic intensification, although estimate confidence intervals overlapped with zero (Figure 4).

## 4 DISCUSSION

Our TBI analysis revealed a set of seven sites with substantial change in community composition over time. Further exploratory analysis using occurrence data rather than abundance data revealed a drop in the number of sites signaling significant change pre‐Holm correction for multiple testing. This result may indicate species have already been lost prior to the fish surveys in 2001, making fluctuations in abundance of remaining species more common and influential in our TBI analysis. The different results in using abundance over occurrence data also indicate some sites may show signs of local extinction debt, where poor conditions reduce abundances, but the species continues to persist at the site for the time being (Figueiredo et al., 2019). The TBI is a useful metric for identifying sites with large community change and can subsequently be used in further modeling to understand differences in drivers between gains and losses of species of abundance‐per‐species (Legendre, 2019; Legendre & Condit, 2019). Further analysis of abundance trends related specifically to gains and losses may reveal underlying community dynamics related to extinction and colonization patterns (Carvalho et al., 2012).

The type of change differed between the high TBI sites, with three sites showing losses and four sites showing gains overall, one high TBI site gaining the invasive round goby, and two high TBI sites gaining green sunfish. The observed 4:3 ratio of gains to losses is the opposite direction of our prediction of more losses than gains. The TBI is determined by change in species presence and/or species abundance; we used abundance‐per‐species due to availability of count data. While sites indicate overall gains or losses, investigating individual species change is important to identify whether gains are gains in species or abundance, and whether gains are native or non‐native species. Gains in native species may be reflective of recovery due to restoration efforts but gains in non‐native species be indicative of broader invasion dynamics ongoing in urban regions. Urban regions are known to have higher prevalence of invasive species (Padayachee et al., 2017); thus, we may expect TBI to increase through time if invasive species presence and abundance increase, which can have an immediate impact on the interpretation of biodiversity metrics, and a longer‐term impact on local biodiversity through changing community dynamics (Gaertner et al., 2017). For example, round goby invasion of tributaries in the Great Lakes region is of concern, as round goby have been found to impact native species through competition for resources and predation on early life stages of native species (Poos et al., 2010). Such competition and predation by non‐native round goby may further impact already declining species by further reducing the abundance of native species, a theory supported by the results of Kindree et al. (2023).

Comparing change in individual species abundances across the study region, each individual watershed, and each stream order revealed little significant change in individual species abundances when corrected for multiple comparison testing. This result may be due to diverse spatial patterns of urbanization across the region causing site‐specific legacy effects (Perring et al., 2016; Wohl, 2019), and the inherent dendritic nature of rivers creating heterogeneous habitat conditions between sites (Benda et al., 2004). Spatial patterns of urbanization may differ among sites due to time since land‐use change, proximity to direct land‐use change, or type of land‐use change occurring within a watershed. For example, some sites further downstream see little change in upstream catchment land use as they have been urbanized for over 100 years, whereas some sites closer to headwaters see higher relative levels of land‐use change as headwaters are relatively less urbanized areas than downstream sites in the study region (Figure S2). Such site specificity may obscure site‐level change when aggregated to a broader spatial extent. While inconvenient for making broader‐scale conclusions on drivers of change in species abundances, this difference suggests metrics like the TBI are useful for identifying individual exceptional change sites within a broader urban matrix which may become of management interest.

The TBI can be scaled up to a higher level of biological organization than species (Legendre, 2019). While the focus of the current paper was species‐level biodiversity change, higher levels of biological organization such as family, or other classifications based on thermal guild or functional traits can be used in future studies (Austen et al., 1994; Benoit et al., 2021). Guild level analyses may reveal broader patterns of change which can be obscured by species‐level investigation. Our results suggest thermal guilds may be an important categorization to consider, as we found one high TBI site shifted from a cooler‐water fish dominated community to a warm‐water fish dominated community (EC010WM). It is possible land‐use change upstream of this site contributed to changes in water temperature, as the site saw anthropogenic land intensification directly upstream, along with small pockets of woodland and wetland loss (Figure 3).

While our temporal beta diversity analysis revealed gains and losses in fish biodiversity across the study sites, our chosen potential drivers of fish community gains and losses had low explanatory power. Model‐averaged predictor‐estimate confidence intervals overlapped with zero for almost all estimates and reported relatively low *R* ^2^ values (Figure 4; Table 2). While calculating land‐use change can be done consistently over large spatial extents through remote sensing, fine‐scale variation can be obscured since land use is a proxy encapsulating many urban changes, and this may have contributed to our results. Finer geographic scale like local upstream catchment at smaller distance buffers or riparian zone land use may reveal stronger relationships to biodiversity indices; however, we were interested in cumulative upstream land‐use impacts at the entire upstream catchment scale. Degree of land‐use change may have fallen below thresholds previously found to explain ecological change. For example, while thresholds for land‐use change tend to be geographically specific, they were found to range from 1% to 12% for urbanization and 2% to 37% for agriculture in a recent global study (Chen & Olden, 2020). The effects of land use may also be statistically stronger when considering factors such as habitat composition. While data to this scale are often not available due to collection limitations through time, it is possible finer‐scale changes in habitat may be stronger drivers of biodiversity change (Chase et al., 2018). Additionally, it is possible physical variables like change in water temperature, may explain some variation (Jarvie & Jackson, 2022), as one site of interest appears to show a change in thermal community composition. As suggested by Kuczynski et al. (2018), including predictors such as specific pollutants or flow may also improve explanation of variation within sites through time. As temporal datasets mature and ecological monitoring continues, analyzing trends over time periods longer than the 15 years analyzed in the current study may reveal stronger relationships between drivers of biodiversity change if time lags are present (Watts et al., 2020). Ultimately, while curating temporal biotic datasets is of the utmost importance, collecting physical and chemical datasets alongside biological data will aid in future explanatory modeling efforts.

The value of open‐source data gathered through standardized sampling methods in long‐term monitoring programs should not be underestimated. Such long‐term datasets provide the basis to answer numerous ecological and conservation‐oriented questions. Long‐term datasets are particularly valuable in regions experiencing ongoing land‐use change and because we cannot logistically (nor ethically) plan and implement the corresponding large‐scale experimental studies that would allow testing of these ecological responses (i.e., experiments to test the effects of environmental stressors common to urbanized regions at the scale of urban centers). The evolution of the “sustainable city” from the “industrial” and “sanitary” city makes for an interesting comparison of ecological change over time (Kaushal et al., 2015). The approach in this paper can be used to examine ecological change, whether due to further urbanization or restoration. Future studies can include more land‐use categories where land is restored to more natural land cover, unlike the current study where only land use change in the direction of further anthropogenic intensification was included. However, such studies will only be possible with continued monitoring of ecosystems through time using standardized approaches such as those used in long‐term monitoring programs. Considering the ongoing “Decade on Restoration” and the recent 2023 Kunming‐Montreal GBF, the collection of data to inform biodiversity‐inclusive planning and the funding of research to analyze pre‐existing long‐term datasets must be a regional and national priority to meet international biodiversity goals.

## AUTHOR CONTRIBUTIONS

**Lauren Lawson:** Conceptualization (lead); data curation (lead); formal analysis (lead); methodology (lead); visualization (lead); writing – original draft (lead); writing – review and editing (lead). **Christopher B. Edge:** Conceptualization (equal); data curation (supporting); methodology (equal); resources (equal); supervision (lead); writing – original draft (supporting); writing – review and editing (equal). **Marie‐Josée Fortin:** Conceptualization (equal); methodology (supporting); writing – review and editing (supporting). **Donald A. Jackson:** Conceptualization (equal); methodology (supporting); supervision (lead); writing – review and editing (supporting).

## CONFLICT OF INTEREST STATEMENT

The authors declare no conflicts of interest.

## Supporting information


Data S1

## Data Availability

Data and analysis code are available at https://github.com/Lauren2015L/Toronto‐fish‐community‐2023.

## References

[ece310845-bib-0001] Arnold, C. L. , & Gibbons, C. J. (1996). Impervious surface coverage: The emergence of a key environmental indicator. Journal of the American Planning Association, 62, 243–258.

[ece310845-bib-0002] Austen, D. J. , Bayley, P. B. , & Menzel, B. W. (1994). Importance of the guild concept to fisheries research and management. Fisheries, 19, 12–20.

[ece310845-bib-0003] Barton, K. (2022). Mumin: multi‐model inference. R .

[ece310845-bib-0004] Baselga, A. (2008). Determinants of species richness, endemism and turnover in European longhorn beetles. Ecography, 31, 263–271.

[ece310845-bib-0005] Baselga, A. (2010). Partitioning the turnover and nestedness components of beta diversity: Partitioning beta diversity. Global Ecology and Biogeography, 19, 134–143.

[ece310845-bib-0006] Benda, L. , Poff, N. L. , Miller, D. , Dunne, T. , Reeves, G. , Pess, G. , & Pollock, M. (2004). The network dynamics hypothesis: How channel networks structure riverine habitats. Bioscience, 54, 413.

[ece310845-bib-0007] Benoit, D. M. , Jackson, D. A. , & Chu, C. (2021). Partitioning fish communities into guilds for ecological analyses: An overview of current approaches and future directions. Canadian Journal of Fisheries and Aquatic Sciences, 78, 984–993.

[ece310845-bib-0008] Bonnell, J. L. (2014). Reclaiming the Don: An environmental history of Toronto's Don river valley. University of Toronto Press.

[ece310845-bib-0009] Brown, B. L. , & Swan, C. M. (2010). Dendritic network structure constrains metacommunity properties in riverine ecosystems. Journal of Animal Ecology, 79, 571–580. 20180874 10.1111/j.1365-2656.2010.01668.x

[ece310845-bib-0010] Burnham, K. P. , Anderson, D. R. , & Huyvaert, K. P. (2011). Erratum to: Aic model selection and multimodel inference in behavioral ecology: Some background, observations, and comparisons. Behavioral Ecology and Sociobiology, 65, 415.

[ece310845-bib-0011] Careless, J. M. S. (1984). Toronto to 1918: An illustrated history. J. Lorimer & Co., National Museum of Man, National Museums of Canada.

[ece310845-bib-0012] Carvalho, J. C. , Cardoso, P. , & Gomes, P. (2012). Determining the relative roles of species replacement and species richness differences in generating beta‐diversity patterns: Partitioning beta diversity. Global Ecology and Biogeography, 21, 760–771.

[ece310845-bib-0013] CBD . (2022). Kunming‐Montreal global biodiversity framework draft decision. United Nations COP15, United Nations conference of the parties to the convention on biological diversity. CBD.

[ece310845-bib-0014] Chase, J. M. , Mcgill, B. J. , Mcglinn, D. J. , May, F. , Blowes, S. A. , Xiao, X. , Knight, T. M. , Purschke, O. , & Gotelli, N. J. (2018). Embracing scale‐dependence to achieve a deeper understanding of biodiversity and its change across communities. Ecology Letters, 21, 1737–1751. 30182500 10.1111/ele.13151

[ece310845-bib-0015] Chen, K. , & Olden, J. D. (2020). Threshold responses of riverine fish communities to land use conversion across regions of the world. Global Change Biology, 26, 4952–4965. 32564461 10.1111/gcb.15251

[ece310845-bib-0016] Choy, M. , Lawrie, D. , & Edge, C. B. (2018). Measuring 30 years of improvements to aquatic connectivity in the greater Toronto area. Aquatic Ecosystem Health & Management, 21, 342–351.

[ece310845-bib-0063] Cote, D. , Kehler, D. G. , Bourne, C. , & Wiersma, Y. F. (2009). A new measure of longitudinal connectivity for stream networks. Landscape Ecology, 24(1), 101–113.

[ece310845-bib-0017] Damgaard, C. (2019). A critique of the space‐for‐time substitution practice in community ecology. Trends in Ecology & Evolution, 34, 416–421. 30824195 10.1016/j.tree.2019.01.013

[ece310845-bib-0018] Dejong, T. M. (1975). A comparison of three diversity indices based on their components of richness and evenness. Oikos, 26, 222.

[ece310845-bib-0019] Dray, S. , Bauman, D. , Blanchet, G. , Borcard, D. , Clappe, S. , Guenard, G. , Jombart, T. , Larocque, G. , Legendre, P. , Madi, N. , & Wagner, H. H. (2021). Adespatial: Multivariate multiscale spatial analysis .

[ece310845-bib-0020] Edge, C. B. , Fortin, M.‐J. , Jackson, D. A. , Lawrie, D. , Stanfield, L. , & Shrestha, N. (2017). Habitat alteration and habitat fragmentation differentially affect beta diversity of stream fish communities. Landscape Ecology, 32, 647–662.

[ece310845-bib-0021] Figueiredo, L. , Krauss, J. , Steffan‐Dewenter, I. , & Sarmento Cabral, J. (2019). Understanding extinction debts: Spatio–temporal scales, mechanisms and a roadmap for future research. Ecography, 42, 1973–1990.

[ece310845-bib-0022] Foster, D. , Swanson, F. , Aber, J. , Burke, I. , Brokaw, N. , Tilman, D. , & Knapp, A. (2003). The importance of land‐use legacies to ecology and conservation. Bioscience, 53, 77.

[ece310845-bib-0023] Gaertner, M. , Wilson, J. R. U. , Cadotte, M. W. , Macivor, J. S. , Zenni, R. D. , & Richardson, D. M. (2017). Non‐native species in urban environments: Patterns, processes, impacts and challenges. Biological Invasions, 19, 3461–3469.

[ece310845-bib-0024] Haidvogl, G. (2018). Historic milestones of human river uses and ecological impacts. In S. Schmutz & J. Sendzimir (Eds.), Riverine ecosystem management (pp. 19–39). Springer International Publishing.

[ece310845-bib-0025] Harrison, X. A. , Donaldson, L. , Correa‐Cano, M. E. , Evans, J. , Fisher, D. N. , Goodwin, C. E. D. , Robinson, B. S. , Hodgson, D. J. , & Inger, R. (2018). A brief introduction to mixed effects modelling and multi‐model inference in ecology. PeerJ, 6, e4794. 29844961 10.7717/peerj.4794 PMC5970551

[ece310845-bib-0026] Hein, C. (2016). Port cities and urban waterfronts: How localized planning ignores water as a connector. WIREs Water, 3, 419–438.

[ece310845-bib-0027] Holm, S. (1979). A simple sequentially rejective multiple test procedure. Scandinavian Journal of Statistics, 6, 65–70.

[ece310845-bib-0028] Hughes, B. B. , Beas‐Luna, R. , Barner, A. K. , Brewitt, K. , Brumbaugh, D. R. , Cerny‐Chipman, E. B. , Close, S. L. , Coblentz, K. E. , De Nesnera, K. L. , Drobnitch, S. T. , Figurski, J. D. , Focht, B. , Friedman, M. , Freiwald, J. , Heady, K. K. , Heady, W. N. , Hettinger, A. , Johnson, A. , Karr, K. A. , … Carr, M. H. (2017). Long‐term studies contribute disproportionately to ecology and policy. Bioscience, 67, 271–281.

[ece310845-bib-0029] Jarvie, M. , & Jackson, D. A. (2022). Weighted stream temperature tolerance index is insensitive to changes in stream fish composition. Freshwater Science, 41, 386–397.

[ece310845-bib-0030] Jost, l. (2007). Partitioning diversity into independent alpha and beta components. Ecology, 88, 2427–2439. 18027744 10.1890/06-1736.1

[ece310845-bib-0031] Jost, l. (2010). The relation between evenness and diversity. Diversity, 2, 207–232.

[ece310845-bib-0032] Jung, M. , Rowhani, P. , & Scharlemann, J. P. W. (2019). Impacts of past abrupt land change on local biodiversity globally. Nature Communications, 10, 5474. 10.1038/s41467-019-13452-3 PMC6888856 31792206

[ece310845-bib-0033] Kaushal, S. , Mcdowell, W. , Wollheim, W. , Johnson, T. , Mayer, P. , Belt, K. , & Pennino, M. (2015). Urban evolution: The role of water. Water, 7, 4063–4087.

[ece310845-bib-0034] Khor, N. (2022). World cities report 2022: Envisaging the future of cities. United Nations Human Settlements Programme (UN‐Habitat).

[ece310845-bib-0035] Kindree, M. M. , Jones, N. E. , & Mandrak, N. E. (2023). Competitive interactions between invasive round goby and native white sucker in streams. Canadian Journal of Fisheries and Aquatic Sciences, 80, 978.

[ece310845-bib-0036] Koleff, P. , Gaston, K. J. , & Lennon, J. J. (2003). Measuring beta diversity for presence‐absence data. Journal of Animal Ecology, 72, 367–382.

[ece310845-bib-0037] Kondratyeva, A. , Knapp, S. , Durka, W. , Kühn, I. , Vallet, J. , Machon, N. , Martin, G. , Motard, E. , Grandcolas, P. , & Pavoine, S. (2020). Urbanization effects on biodiversity revealed by a two‐scale analysis of species functional uniqueness vs. redundancy. Frontiers in Ecology and Evolution, 8, 73.

[ece310845-bib-0038] Kuczynski, L. , Legendre, P. , & Grenouillet, G. (2018). Concomitant impacts of climate change, fragmentation and non‐native species have led to reorganization of fish communities since the 1980s. Global Ecology and Biogeography, 27, 213–222.

[ece310845-bib-0039] Legendre, P. (2019). A temporal beta‐diversity index to identify sites that have changed in exceptional ways in space–time surveys. Ecology and Evolution, 9, 3500–3514. 30962908 10.1002/ece3.4984 PMC6434560

[ece310845-bib-0040] Legendre, P. , & Condit, R. (2019). Spatial and temporal analysis of beta diversity in the Barro Colorado Island forest dynamics plot, Panama. Forest Ecosystems, 6, 7.

[ece310845-bib-0041] Lyashevska, O. , & Farnsworth, K. D. (2012). How many dimensions of biodiversity do we need? Ecological Indicators, 18, 485–492.

[ece310845-bib-0042] Magurran, A. E. (2012). Ecological diversity and its measurement. Springer.

[ece310845-bib-0043] Magurran, A. E. (2021). Measuring biological diversity. Current Biology, 31, r1174–r1177. 34637726 10.1016/j.cub.2021.07.049

[ece310845-bib-0044] Mazerolle, M. J. (2020). Aiccmodavg: Model selection and multimodel inference based on (q)aic(c) .

[ece310845-bib-0045] Oksanen, J. , Blanchet, G. , Friendly, M. , Kindt, R. , Legendre, P. , Mcglinn, D. , Minchin, P. R. , O'hara, R. B. , Simpson, G. L. , Solymos, P. , Stevens, H. H. , Szoecs, E. , & Wagner, H. (2020). Vegan: Community ecology package .

[ece310845-bib-0046] OMNRF . (2019). Southern Ontario land resource information system (SOLRIS) version 3.0: Data specifications (p. 40). Ministry Of Natural Resources and Forestry Science and Research Branch.

[ece310845-bib-0047] Padayachee, A. L. , Irlich, U. M. , Faulkner, K. T. , Gaertner, M. , Procheş, Ş. , Wilson, J. R. U. , & Rouget, M. (2017). How do invasive species travel to and through urban environments? Biological Invasions, 19, 3557–3570.

[ece310845-bib-0048] Perring, M. P. , De Frenne, P. , Baeten, L. , Maes, S. L. , Depauw, L. , Blondeel, H. , Carón, M. M. , & Verheyen, K. (2016). Global environmental change effects on ecosystems: The importance of land‐use legacies. Global Change Biology, 22, 1361–1371. 26546049 10.1111/gcb.13146

[ece310845-bib-0049] Poos, M. , Dextrase, A. J. , Schwalb, A. N. , & Ackerman, J. D. (2010). Secondary invasion of the round goby into high diversity great lakes tributaries and species at risk hotspots: Potential new concerns for endangered freshwater species. Biological Invasions, 12, 1269–1284.

[ece310845-bib-0050] R Core Team . (2022). R: A language and environment for statistical computing. Foundation for Statistical Computing.

[ece310845-bib-0051] Robichaud, C. D. , & Rooney, R. C. (2022). Invasive grass causes biotic homogenization in wetland birds in a Lake Erie coastal marsh. Hydrobiologia, 849, 3197–3212.

[ece310845-bib-0052] Stanfield, l. (2010). Ontario stream assessment protocol (p. 376). Ontario Ministry Of Natural Resources.

[ece310845-bib-0053] Symonds, M. R. E. , & Moussalli, A. (2011). A brief guide to model selection, multimodel inference and model averaging in behavioural ecology using Akaike's information criterion. Behavioral Ecology and Sociobiology, 65, 13–21.

[ece310845-bib-0054] Trautwein, C. , Schinegger, R. , & Schmutz, S. (2012). Cumulative effects of land use on fish metrics in different types of running waters in Austria. Aquatic Sciences, 74, 329–341. 25983526 10.1007/s00027-011-0224-5 PMC4425263

[ece310845-bib-0055] TRCA . (2022). Regional watershed monitoring program .

[ece310845-bib-0056] Turner, B. L. , Lambin, E. F. , & Reenberg, A. (2007). The emergence of land change science for global environmental change and sustainability. Proceedings of the National Academy of Sciences of the United States of America, 104, 20666–20671. 18093934 10.1073/pnas.0704119104 PMC2409212

[ece310845-bib-0057] Turner, W. , Rondinini, C. , Pettorelli, N. , Mora, B. , Leidner, A. K. , Szantoi, Z. , Buchanan, G. , Dech, S. , Dwyer, J. , Herold, M. , Koh, L. P. , Leimgruber, P. , Taubenboeck, H. , Wegmann, M. , Wikelski, M. , & Woodcock, C. (2015). Free and open‐access satellite data are key to biodiversity conservation. Biological Conservation, 182, 173–176.

[ece310845-bib-0058] Walsh, C. J. , Roy, A. H. , Feminella, J. W. , Cottingham, P. D. , Groffman, P. M. , & Morgan, R. P. (2005). The urban stream syndrome: Current knowledge and the search for a cure. Journal of the North American Benthological Society, 24, 706–723.

[ece310845-bib-0059] Ward, J. , & Stanford, J. (1983). The serial discontinuity concept of lotic ecosystems. In T. D. Fontaine & S. M. Bartell (Eds.), Dynamics of lotic ecosystems (pp. 29–42). Ann Arbor Science.

[ece310845-bib-0060] Watts, K. , Whytock, R. C. , Park, K. J. , Fuentes‐Montemayor, E. , Macgregor, N. A. , Duffield, S. , & Mcgowan, P. J. K. (2020). Ecological time lags and the journey towards conservation success. Nature Ecology & Evolution, 4, 304–311. 31988448 10.1038/s41559-019-1087-8

[ece310845-bib-0061] Wohl, E. (2019). Forgotten legacies: Understanding and mitigating historical human alterations of river corridors. Water Resources Research, 55, 5181–5201.

[ece310845-bib-0062] WWF . (2020). Living planet report 2020 – Bending the curve of biodiversity loss .

