# Supplementary material for: Temporal change in urban fish biodiversity—Gains, losses, and drivers of change
Source: Ecol Evol. 2024 Feb 6;14(2):e10845. doi: 10.1002/ece3.10845 (PMC10847622; doi:10.1002/ece3.10845)
Supplement: Supplementary file 1 — Data S1 [file ECE3-14-e10845-s001.docx]

**Supplementary Figures**


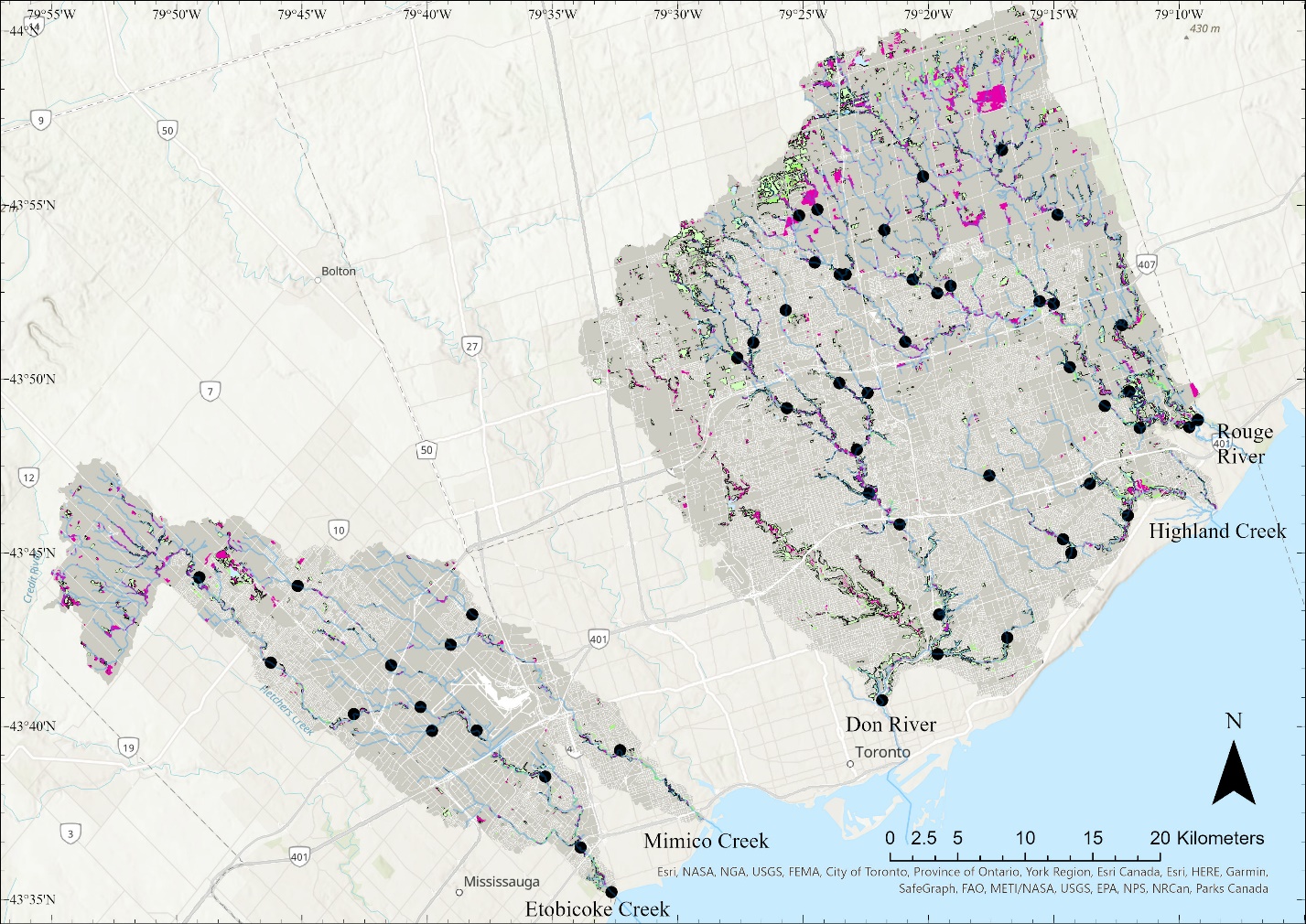


Figure S1: Landcover T1. Grey indicates anthropogenic, green represents woodland, and purple indicates wetland. Black points represent fish sampling sites.


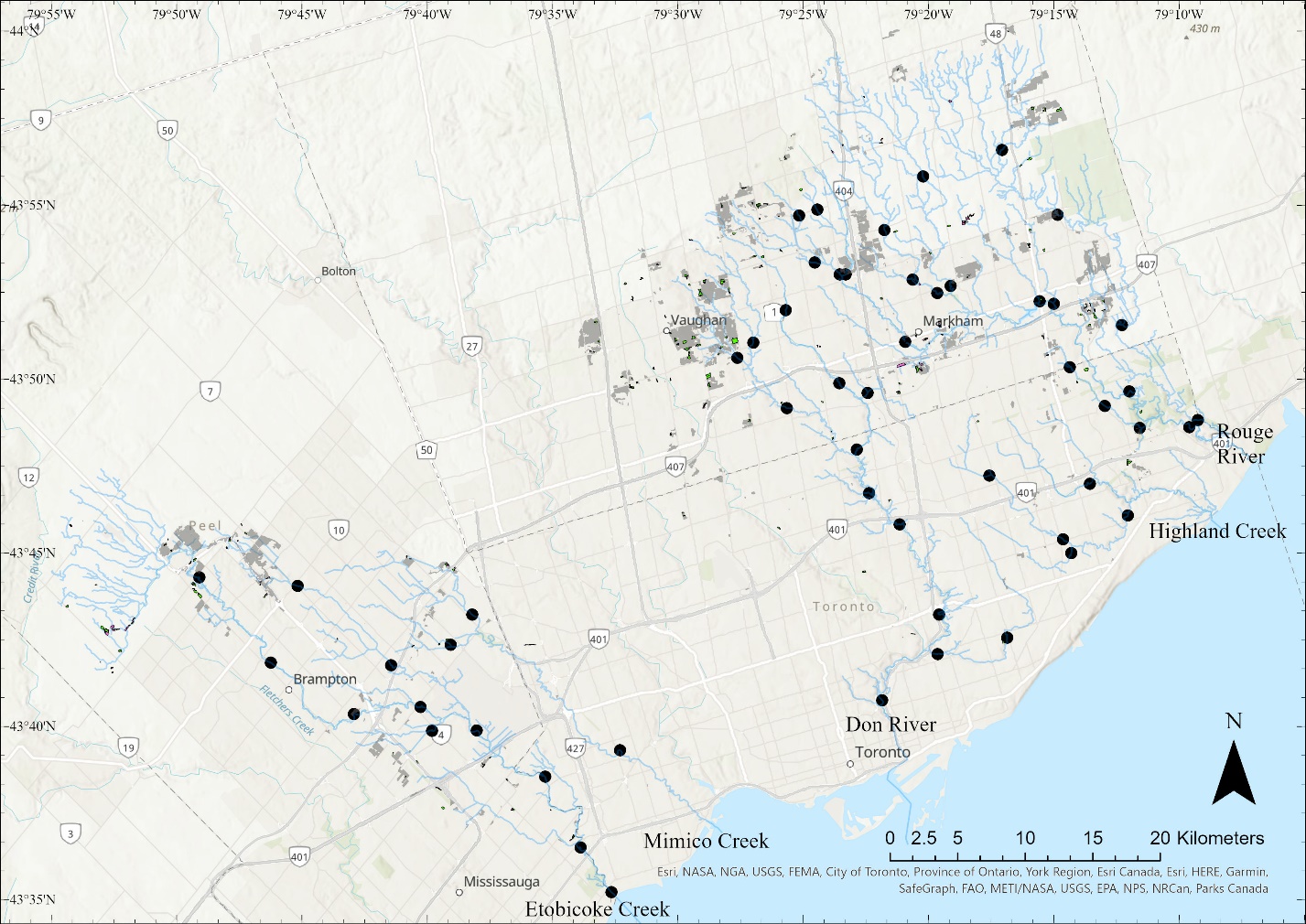


Figure S2: Landcover change T1-T2. Grey indicates anthropogenic intensification, green indicates woodland loss, purple indicates wetland loss. Black points represent fish sampling sites.


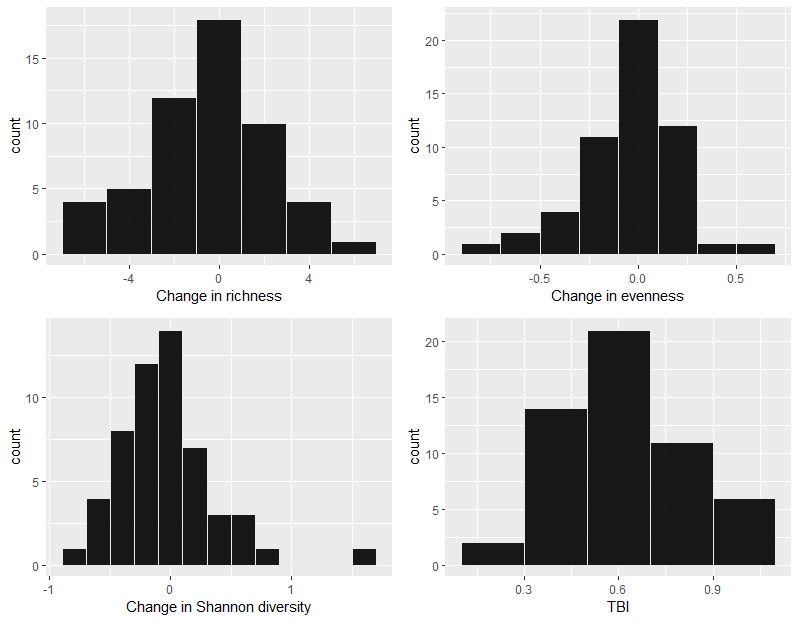


Figure S3: Histogram of change in species richness, change in Pielou’s evenness, change in Shannon diversity, and the TBI. Bin-width for richness is 2, bin-width for Pielou’s evenness, Shannon diversity, and the TBI is .2

**Supplementary Tables:**

Table S1: Species decision-making table for cleaning raw fish community data. All spelling errors were corrected at the species level and species were combined by year per site. Species were grouped by station and year, and then aggregated based on common_name. Don River DN011 removed in 2003 because randomly extra sampled that year while the remaining Don River sites were sampled in 2002 causing duplicates for DN011. ‘Common_Name’ column reflects corresponding terminology of the column ‘Common_Name’ in the raw dataset, we recognize some entries are not the true common name in the raw dataset collected given level of classification and note below where this is corrected when possible.

| ‘Common_  Name’ label in raw data | Instances and abundances | | Potential | Decision | Action |
| --- | --- | --- | --- | --- | --- |
|  | T1 | T2 |  |  |  |
| Etheostoma | DN001 (n = 8)  DN005 (n = 11)  DN006 (n = 49)  DN008 (n 13  DN018 (n = 2)  DN021 (n = 4)  DN022 (n = 3)  RG001 WM (n = 3)  RG008WM (n = 13) | 0 | Darters | Only Johnny Darter occurrence on Don River sites, replaced with Johnny Darter on Don River  Rouge site contain 3 darter species so removed observation as unable to confidently sort | Changed or removed |
| Catostomus sp. | DN009 (n = 28)  DN010 (n = 12)  DN021 (n = 4)  DN022 (n = 10)  RG009 (n = 1)  RG001WM  (n = 6) | 0 | Sucker | DN009, DN010, DN022, RG009 changed to white sucker.  DN021WM removed given could be either white sucker or northern hog sucker | Changed or removed |
| UNKNOWN (ANY OR ALL FISH SPECIES) | EC007 (n = 27)  EC009 (n = 50)  DN013 ( n = 2) | RG009  (n = 1) | NA | Removed all | Removed |
| Catostomidae | RG009WM  (n = 1) | RG001WM (n = 6) | Sucker | Changed to white sucker given site history | Changed |
| Petromyzontidae | RG019WM  (n = 4)  RG023WM  (n = 1) | 0 | Lampreys | Replace with American Brook lamprey | Changed |
| Lepomis sp. | RG023 (n = 1) | 0 | Sunfishes | Could be different species. Replace with pumpkinseed because only sunfish species noted in the Rouge system within this dataset | Changed |
| Green sunfish x Pumpkinseed | 0 | EC010WM  (n = 24)  DN021WM (n = 1) | Sunfishes | Hybrids | Leave as is |
| Salmon & Trout subfamily | 0 | n=2 (RG006)  n = 1 (RG007) | NA | Most likely a rainbow trout at the rg006 site, only rainbow trout have been seen previously (in 2009) but only 1 observation in entire dataset so removed | Removed |
| Cyprinidae | NA | NA | NA | Changed to Leuciscidae given more recent change to classification system | Changed name |
| Johnny/Tesselated Darter | 0 | N = 5  EC001  N = 10  EC002 | NA | Could be either species in sites | Removed |
| Centrarchidae sp. | RG008WM  (N = 1)  RG014WM  (n = 1) | RG009WM  (n = 1) | Sunfishes | Likely pumpkinseed for these sites, change RG008 and RG014WM to pumpkin seed | Changed |

Table S2: Decisions for categorizing SOLRIS land cover groups at T1 (2000). Urban represents urbanized or significantly anthropogenically altered land including cropland and impervious land.

| Solris landcover t1 category | Categorization |
| --- | --- |
| Built-up area impervious | Anthropogenic |
| Built-up area pervious | Anthropogenic |
| Extraction | Anthropogenic |
| Hedge rows | Anthropogenic |
| Transportation | Anthropogenic |
| Annual crop | Anthropogenic |
| Mixed crop | Anthropogenic |
| Perennial crop | Anthropogenic |
| Idle land | Anthropogenic |
| Plantations - tree cultiv | Anthropogenic |
| Bog | Wetland |
| Fen | Wetland |
| Marsh | Wetland |
| Swamp | Wetland |
| Coniferous forest | Woodland |
| Deciduous forest | Woodland |
| Forest | Woodland |
| Mixed forest | Woodland |
| Open water | Not included |
| Shallow water | Not included |

Table S3: Decisions for categorizing SOLRIS land cover groups for change in land cover between T1 and T2 (2015).

| Wetland loss | Woodland loss | Anthropogenic transition |
| --- | --- | --- |
| Wetland to built up area-impervious | Woodland to Built Up Area-Impervious | Built Up Area-Impervious to Built Up Area-Impervious |
| Wetland to built up area-pervious | Woodland to Built Up Area-Pervious | Built Up Area-Pervious to Built Up Area-Impervious |
| Wetland to extraction-aggregate | Woodland to Extraction | Hedgerow to Built Up Area-Impervious |
| Wetland to tilled | Woodland to Extraction-Aggregate | Hedgerow to Extraction-Aggregate |
| Wetland to transportation | Woodland to Tilled | Plantation to Built Up Area-Impervious |
| Wetland to undifferentiated | Woodland to Transportation | Tilled to Built Up Area-Impervious |
|  | Woodland to Undifferentiated | Tilled to Built Up Area-Pervious |
|  |  | Tilled to Extraction-Aggregate |
|  |  | Undifferentiated to Built Up Area-Impervious |
|  |  | Undifferentiated to Built Up Area-Pervious |
|  |  | Undifferentiated to Extraction |
|  |  | Undifferentiated to Extraction-Aggregate |
|  |  | Undifferentiated to Transportation |

## Table S4: TBI results per fish sampling site for abundance data. SIGNIF indicates pre-correction significance identification at a threshold of p<0.05.

| Station | TBI | p.TBI | p.holm | Losses | Gains | Signif | Change |
| --- | --- | --- | --- | --- | --- | --- | --- |
| DN001WM | 0.659 | 0.458 | 1.000 | 0.147 | 0.512 |  | + |
| DN002WM | 0.295 | 0.957 | 1.000 | 0.045 | 0.250 |  | + |
| DN003WM | 0.818 | 0.146 | 1.000 | 0.152 | 0.667 |  | + |
| DN005WM | 0.717 | 0.323 | 1.000 | 0.036 | 0.681 |  | + |
| DN006WMb | 0.531 | 0.713 | 1.000 | 0.358 | 0.173 |  | - |
| DN007WM | 0.370 | 0.908 | 1.000 | 0.027 | 0.342 |  | + |
| DN008WM | 0.388 | 0.890 | 1.000 | 0.077 | 0.311 |  | + |
| DN009WMb | 0.758 | 0.243 | 1.000 | 0.303 | 0.455 |  | + |
| DN010WMb | 0.906 | 0.035 | 1.000 | 0.895 | 0.012 | * | - |
| DN011WM | 0.960 | 0.011 | 0.680 | 0.100 | 0.860 | * | + |
| DN021WM | 0.389 | 0.890 | 1.000 | 0.106 | 0.282 |  | + |
| DN022WM | 0.630 | 0.498 | 1.000 | 0.143 | 0.487 |  | + |
| DN023WM | 0.456 | 0.823 | 1.000 | 0.016 | 0.440 |  | + |
| EC001WM | 0.914 | 0.032 | 1.000 | 0.802 | 0.112 | * | - |
| EC002WM | 0.410 | 0.870 | 1.000 | 0.264 | 0.146 |  | - |
| EC004WM | 0.652 | 0.459 | 1.000 | 0.039 | 0.613 |  | + |
| EC005WM | 0.476 | 0.793 | 1.000 | 0.353 | 0.122 |  | - |
| EC006WM | 0.807 | 0.159 | 1.000 | 0.267 | 0.541 |  | + |
| EC007WM | 0.625 | 0.526 | 1.000 | 0.188 | 0.438 |  | + |
| EC008WM | 0.572 | 0.622 | 1.000 | 0.020 | 0.553 |  | + |
| EC009WM | 0.674 | 0.421 | 1.000 | 0.604 | 0.070 |  | - |
| EC010WM | 0.890 | 0.049 | 1.000 | 0.165 | 0.726 | * | + |
| EC011WM | 0.600 | 0.576 | 1.000 | 0.084 | 0.516 |  | + |
| EC012WM | 0.532 | 0.712 | 1.000 | 0.149 | 0.383 |  | + |
| HL003WM | 0.563 | 0.648 | 1.000 | 0.162 | 0.401 |  | + |
| HL005WM | 0.928 | 0.024 | 1.000 | 0.052 | 0.876 | * | + |
| HL009WM | 0.716 | 0.326 | 1.000 | 0.043 | 0.673 |  | + |
| HL010WM | 0.628 | 0.512 | 1.000 | 0.030 | 0.598 |  | + |
| HL011WM | 0.696 | 0.373 | 1.000 | 0.283 | 0.413 |  | + |
| MM002WM | 0.918 | 0.030 | 1.000 | 0.163 | 0.755 | * | + |
| MM004WM | 0.333 | 0.929 | 1.000 | 0.250 | 0.083 |  | - |
| MM005WM | 0.846 | 0.102 | 1.000 | 0.000 | 0.846 |  | + |
| RG001WM | 0.670 | 0.419 | 1.000 | 0.470 | 0.200 |  | - |
| RG002WM | 0.333 | 0.934 | 1.000 | 0.022 | 0.311 |  | + |
| RG003WM | 0.679 | 0.404 | 1.000 | 0.069 | 0.611 |  | + |
| RG004WM | 0.255 | 0.970 | 1.000 | 0.176 | 0.080 |  | - |
| RG005WM | 0.801 | 0.157 | 1.000 | 0.077 | 0.724 |  | + |
| RG006WM | 0.444 | 0.838 | 1.000 | 0.191 | 0.253 |  | + |
| RG007WM | 0.452 | 0.827 | 1.000 | 0.309 | 0.143 |  | - |
| RG009WM | 0.844 | 0.108 | 1.000 | 0.029 | 0.815 |  | + |
| RG010WM | 0.645 | 0.484 | 1.000 | 0.161 | 0.485 |  | + |
| RG011WM | 0.544 | 0.675 | 1.000 | 0.485 | 0.058 |  | - |
| RG012WMb | 0.466 | 0.806 | 1.000 | 0.411 | 0.055 |  | - |
| RG015WM | 0.349 | 0.923 | 1.000 | 0.153 | 0.195 |  | + |
| RG016WM | 0.861 | 0.075 | 1.000 | 0.851 | 0.010 |  | - |
| RG017WM | 0.662 | 0.444 | 1.000 | 0.603 | 0.059 |  | - |
| RG018WM | 0.475 | 0.800 | 1.000 | 0.220 | 0.256 |  | + |
| RG019WM | 0.938 | 0.017 | 1.000 | 0.935 | 0.003 | * | - |
| RG021WM | 0.641 | 0.490 | 1.000 | 0.582 | 0.059 |  | - |
| RG022WM | 0.569 | 0.643 | 1.000 | 0.501 | 0.068 |  | - |
| RG023WM | 0.706 | 0.351 | 1.000 | 0.692 | 0.014 |  | - |
| RG024WM | 0.520 | 0.728 | 1.000 | 0.480 | 0.040 |  | - |
| RG025WM | 0.550 | 0.665 | 1.000 | 0.125 | 0.425 |  | + |
| RG026WM | 0.495 | 0.767 | 1.000 | 0.049 | 0.447 |  | + |

## Table S5: TBI results per fish sampling site for occurrence data. SIGNIF indicates pre-correction significance identification at a threshold of p<0.05.

| Station | TBI | p.TBI | p.holm | Losses | Gains | Signif | Change |
| --- | --- | --- | --- | --- | --- | --- | --- |
| DN001WM | 0.500 | 0.202 | 1.000 | 0.333 | 0.167 |  | - |
| DN002WM | 0.200 | 0.888 | 1.000 | 0.200 | 0.000 |  | - |
| DN003WM | 0.200 | 0.883 | 1.000 | 0.000 | 0.200 |  | + |
| DN005WM | 0.167 | 0.931 | 1.000 | 0.083 | 0.083 |  | 0 |
| DN006WMb | 0.167 | 0.925 | 1.000 | 0.000 | 0.167 |  | + |
| DN007WM | 0.200 | 0.886 | 1.000 | 0.200 | 0.000 |  | - |
| DN008WM | 0.231 | 0.819 | 1.000 | 0.000 | 0.231 |  | + |
| DN009WMb | 0.500 | 0.206 | 1.000 | 0.250 | 0.250 |  | 0 |
| DN010WMb | 0.429 | 0.327 | 1.000 | 0.286 | 0.143 |  | - |
| DN011WM | 0.500 | 0.207 | 1.000 | 0.250 | 0.250 |  | 0 |
| DN021WM | 0.500 | 0.203 | 1.000 | 0.083 | 0.417 |  | + |
| DN022WM | 0.231 | 0.823 | 1.000 | 0.077 | 0.154 |  | + |
| DN023WM | 0.111 | 0.969 | 1.000 | 0.000 | 0.111 |  | + |
| EC001WM | 0.667 | 0.040 | 1.000 | 0.417 | 0.250 | * | - |
| EC002WM | 0.455 | 0.275 | 1.000 | 0.091 | 0.364 |  | + |
| EC004WM | 0.333 | 0.607 | 1.000 | 0.200 | 0.133 |  | - |
| EC005WM | 0.375 | 0.470 | 1.000 | 0.188 | 0.188 |  | 0 |
| EC006WM | 0.556 | 0.117 | 1.000 | 0.000 | 0.556 |  | + |
| EC007WM | 0.400 | 0.381 | 1.000 | 0.050 | 0.350 |  | + |
| EC008WM | 0.263 | 0.730 | 1.000 | 0.053 | 0.211 |  | + |
| EC009WM | 0.400 | 0.382 | 1.000 | 0.300 | 0.100 |  | - |
| EC010WM | 0.714 | 0.026 | 1.000 | 0.143 | 0.571 | * | + |
| EC011WM | 0.143 | 0.952 | 1.000 | 0.071 | 0.071 |  | 0 |
| EC012WM | 0.444 | 0.285 | 1.000 | 0.278 | 0.167 |  | - |
| HL003WM | 0.111 | 0.968 | 1.000 | 0.111 | 0.000 |  | - |
| HL005WM | 0.429 | 0.336 | 1.000 | 0.429 | 0.000 |  | - |
| HL009WM | 0.250 | 0.773 | 1.000 | 0.125 | 0.125 |  | 0 |
| HL010WM | 0.333 | 0.612 | 1.000 | 0.111 | 0.222 |  | + |
| HL011WM | 0.600 | 0.084 | 1.000 | 0.400 | 0.200 |  | - |
| MM002WM | 0.600 | 0.082 | 1.000 | 0.200 | 0.400 |  | + |
| MM004WM | 0.500 | 0.209 | 1.000 | 0.250 | 0.250 |  | 0 |
| MM005WM | 0.000 | 1.000 | 1.000 | 0.000 | 0.000 |  | 0 |
| RG001WM | 0.333 | 0.615 | 1.000 | 0.296 | 0.037 |  | - |
| RG002WM | 0.500 | 0.204 | 1.000 | 0.167 | 0.333 |  | + |
| RG003WM | 0.333 | 0.617 | 1.000 | 0.111 | 0.222 |  | + |
| RG004WM | 0.143 | 0.946 | 1.000 | 0.071 | 0.071 |  | 0 |
| RG005WM | 0.286 | 0.691 | 1.000 | 0.071 | 0.214 |  | + |
| RG006WM | 0.455 | 0.275 | 1.000 | 0.091 | 0.364 |  | + |
| RG007WM | 0.200 | 0.891 | 1.000 | 0.000 | 0.200 |  | + |
| RG009WM | 0.474 | 0.209 | 1.000 | 0.211 | 0.263 |  | + |
| RG010WM | 0.467 | 0.232 | 1.000 | 0.067 | 0.400 |  | + |
| RG011WM | 0.300 | 0.618 | 1.000 | 0.200 | 0.100 |  | - |
| RG012WMb | 0.304 | 0.608 | 1.000 | 0.261 | 0.043 |  | - |
| RG015WM | 0.238 | 0.778 | 1.000 | 0.095 | 0.143 |  | + |
| RG016WM | 0.391 | 0.390 | 1.000 | 0.348 | 0.043 |  | - |
| RG017WM | 0.385 | 0.443 | 1.000 | 0.231 | 0.154 |  | - |
| RG018WM | 0.333 | 0.605 | 1.000 | 0.167 | 0.167 |  | 0 |
| RG019WM | 0.529 | 0.148 | 1.000 | 0.471 | 0.059 |  | - |
| RG021WM | 0.333 | 0.610 | 1.000 | 0.238 | 0.095 |  | - |
| RG022WM | 0.238 | 0.779 | 1.000 | 0.190 | 0.048 |  | - |
| RG023WM | 0.300 | 0.621 | 1.000 | 0.200 | 0.100 |  | - |
| RG024WM | 0.467 | 0.231 | 1.000 | 0.333 | 0.133 |  | - |
| RG025WM | 0.200 | 0.882 | 1.000 | 0.000 | 0.200 |  | + |
| RG026WM | 0.500 | 0.206 | 1.000 | 0.167 | 0.333 |  | + |

Table S6: Paired t-test results across the entire watershed

| Species | mean  (T1-T2) | t.stat | p.param | p.perm | p<=0.05 | Sign  (T1-T2) | p.holm.adj |
| --- | --- | --- | --- | --- | --- | --- | --- |
| American Brook Lamprey | 0.204 | 1.848 | 0.035 | 0.063 |  | 1.000 | 1.000 |
| Blacknose Dace | -20.796 | -1.703 | 0.047 | 0.051 |  | -1.000 | 1.000 |
| Bluegill | -0.019 | -1.000 | 0.161 | 0.509 |  | -1.000 | 1.000 |
| Bluntnose Minnow | 0.130 | 0.066 | 0.474 | 0.491 |  | 1.000 | 1.000 |
| Brook Stickleback | 1.907 | 1.437 | 0.078 | 0.095 |  | 1.000 | 1.000 |
| Brook Trout | -0.037 | -1.000 | 0.161 | 0.494 |  | -1.000 | 1.000 |
| Brown Bullhead | -0.185 | -1.564 | 0.062 | 0.139 |  | -1.000 | 1.000 |
| Brown Trout | 0.611 | 1.126 | 0.133 | 0.154 |  | 1.000 | 1.000 |
| Central Mudminnow | -0.019 | -1.000 | 0.161 | 0.488 |  | -1.000 | 1.000 |
| Central Stoneroller | -0.019 | -0.007 | 0.497 | 0.503 |  | -1.000 | 1.000 |
| Common Carp | -0.037 | -0.164 | 0.435 | 0.386 |  | -1.000 | 1.000 |
| Common Shiner | -3.278 | -1.257 | 0.107 | 0.131 |  | -1.000 | 1.000 |
| Creek Chub | 1.296 | 0.386 | 0.350 | 0.348 |  | 1.000 | 1.000 |
| Leuciscidae | -0.574 | -0.355 | 0.362 | 0.471 |  | -1.000 | 1.000 |
| Emerald Shiner | -0.148 | -1.000 | 0.161 | 0.481 |  | -1.000 | 1.000 |
| Fantail Darter | -0.296 | -1.000 | 0.161 | 0.511 |  | -1.000 | 1.000 |
| Fathead Minnow | -5.204 | -1.745 | 0.043 | 0.040 | * | -1.000 | 1.000 |
| Golden Shiner | 0.722 | 1.733 | 0.044 | 0.018 | * | 1.000 | 0.720 |
| Goldfish | 0.278 | 1.235 | 0.111 | 0.127 |  | 1.000 | 1.000 |
| Green Sunfish | -4.963 | -1.051 | 0.149 | 0.028 | * | -1.000 | 1.000 |
| Green Sunfish x Pumpkinseed | -0.463 | -1.042 | 0.151 | 0.262 |  | -1.000 | 1.000 |
| Hornyhead Chub | 0.407 | 1.707 | 0.047 | 0.074 |  | 1.000 | 1.000 |
| Johnny Darter | 1.259 | 0.515 | 0.304 | 0.338 |  | 1.000 | 1.000 |
| Largemouth Bass | -0.074 | -1.659 | 0.051 | 0.126 |  | -1.000 | 1.000 |
| Longnose Dace | -12.963 | -1.402 | 0.083 | 0.091 |  | -1.000 | 1.000 |
| Mottled Sculpin | -1.037 | -1.933 | 0.029 | 0.003 | * | -1.000 | 0.123 |
| Northern Hog Sucker | -0.093 | -0.819 | 0.208 | 0.499 |  | -1.000 | 1.000 |
| Northern Redbelly Dace | 0.241 | 1.081 | 0.142 | 0.259 |  | 1.000 | 1.000 |
| Pumpkinseed | 0.481 | 0.879 | 0.192 | 0.246 |  | 1.000 | 1.000 |
| Rainbow Darter | 1.056 | 0.775 | 0.221 | 0.250 |  | 1.000 | 1.000 |
| Rainbow Trout | 1.648 | 1.452 | 0.076 | 0.092 |  | 1.000 | 1.000 |
| Redside Dace | 0.167 | 1.642 | 0.053 | 0.092 |  | 1.000 | 1.000 |
| Rock Bass | 0.574 | 1.922 | 0.030 | 0.031 | * | 1.000 | 1.000 |
| Rosyface Shiner | 0.019 | 1.000 | 0.161 | 0.472 |  | 1.000 | 1.000 |
| Round Goby | -1.444 | -1.054 | 0.148 | 0.252 |  | -1.000 | 1.000 |
| Sand Shiner | 0.019 | 1.000 | 0.161 | 0.512 |  | 1.000 | 1.000 |
| Smallmouth Bass | 0.167 | 1.000 | 0.161 | 0.213 |  | 1.000 | 1.000 |
| Spotfin Shiner | 0.111 | 1.181 | 0.121 | 0.241 |  | 1.000 | 1.000 |
| Spottail Shiner | 0.278 | 1.320 | 0.096 | 0.129 |  | 1.000 | 1.000 |
| Stonecat | -0.074 | -0.455 | 0.325 | 0.465 |  | -1.000 | 1.000 |

Table S7: Paired *t*-test results for species within each watershed sorted by watershed.

| Watershed | Species | mean  (T1-T2) | t.stat | p.param | p.perm | p<=0.05 | Sign  (T1-T2) | p.holm.adj |
| --- | --- | --- | --- | --- | --- | --- | --- | --- |
| Don | American Brook Lamprey | 0.308 | 1.000 | 0.169 | 0.504 |  | 1.000 | 1.000 |
| Don | Blacknose Dace | -20.692 | -1.420 | 0.091 | 0.096 |  | -1.000 | 1.000 |
| Don | Bluntnose Minnow | 1.385 | 1.000 | 0.169 | 0.498 |  | 1.000 | 1.000 |
| Don | Brown Bullhead | -0.385 | -1.000 | 0.169 | 0.506 |  | -1.000 | 1.000 |
| Don | Common Shiner | -0.308 | -1.477 | 0.083 | 0.249 |  | -1.000 | 1.000 |
| Don | Creek Chub | 3.538 | 0.643 | 0.266 | 0.296 |  | 1.000 | 1.000 |
| Don | Fathead Minnow | -9.308 | -1.185 | 0.129 | 0.101 |  | -1.000 | 1.000 |
| Don | Green Sunfish x Pumpkinseed | -0.077 | -1.000 | 0.169 | 0.506 |  | -1.000 | 1.000 |
| Don | Johnny Darter | 3.692 | 1.781 | 0.050 | 0.060 |  | 1.000 | 0.837 |
| Don | Longnose Dace | -24.231 | -1.252 | 0.117 | 0.096 |  | -1.000 | 1.000 |
| Don | Mottled Sculpin | -3.077 | -1.475 | 0.083 | 0.251 |  | -1.000 | 1.000 |
| Don | Northern Hog Sucker | -0.462 | -1.000 | 0.169 | 0.506 |  | -1.000 | 1.000 |
| Don | Pumpkinseed | -1.000 | -1.079 | 0.151 | 0.246 |  | -1.000 | 1.000 |
| Don | Round Goby | -5.692 | -1.000 | 0.169 | 0.502 |  | -1.000 | 1.000 |
| Don | White Sucker | 6.692 | 2.859 | 0.007 | 0.002 | * | 1.000 | 0.032 |
| Etobicoke | Blacknose Dace | -31.091 | -0.897 | 0.196 | 0.231 |  | -1.000 | 1.000 |
| Etobicoke | Bluntnose Minnow | 3.909 | 0.546 | 0.298 | 0.265 |  | 1.000 | 1.000 |
| Etobicoke | Brook Stickleback | 5.273 | 0.907 | 0.193 | 0.373 |  | 1.000 | 1.000 |
| Etobicoke | Brown Bullhead | -0.364 | -1.000 | 0.170 | 0.498 |  | -1.000 | 1.000 |
| Etobicoke | Central Mudminnow | -0.091 | -1.000 | 0.170 | 0.508 |  | -1.000 | 1.000 |
| Etobicoke | Central Stoneroller | -10.909 | -1.864 | 0.046 | 0.008 | * | -1.000 | 0.184 |
| Etobicoke | Common Shiner | -22.545 | -2.090 | 0.032 | 0.007 | * | -1.000 | 0.158 |
| Etobicoke | Leuciscidae | 1.000 | 0.753 | 0.234 | 0.502 |  | 1.000 | 1.000 |
| Etobicoke | Emerald Shiner | -0.727 | -1.000 | 0.170 | 0.501 |  | -1.000 | 1.000 |
| Etobicoke | Fantail Darter | -1.455 | -1.000 | 0.170 | 0.498 |  | -1.000 | 1.000 |
| Etobicoke | Fathead Minnow | 1.545 | 0.630 | 0.272 | 0.419 |  | 1.000 | 1.000 |
| Etobicoke | Golden Shiner | 3.545 | 1.891 | 0.044 | 0.016 | * | 1.000 | 0.352 |
| Etobicoke | Green Sunfish | -24.364 | -1.056 | 0.158 | 0.030 | * | -1.000 | 0.632 |
| Etobicoke | Green Sunfish x Pumpkinseed | -2.182 | -1.000 | 0.170 | 0.507 |  | -1.000 | 1.000 |
| Etobicoke | Johnny Darter | -8.909 | -1.089 | 0.151 | 0.236 |  | -1.000 | 1.000 |
| Etobicoke | Longnose Dace | -5.636 | -0.166 | 0.436 | 0.458 |  | -1.000 | 1.000 |
| Etobicoke | Northern Hog Sucker | 0.091 | 1.000 | 0.170 | 0.512 |  | 1.000 | 1.000 |
| Etobicoke | Pumpkinseed | 0.727 | 0.393 | 0.351 | 0.308 |  | 1.000 | 1.000 |
| Etobicoke | Rainbow Darter | -0.909 | -1.910 | 0.043 | 0.126 |  | -1.000 | 1.000 |
| Etobicoke | Rock Bass | 0.091 | 1.000 | 0.170 | 0.504 |  | 1.000 | 1.000 |
| Etobicoke | Round Goby | -0.364 | -1.000 | 0.170 | 0.504 |  | -1.000 | 1.000 |
| Etobicoke | Spotfin Shiner | 0.545 | 1.200 | 0.129 | 0.249 |  | 1.000 | 1.000 |
| Etobicoke | Spottail Shiner | 1.364 | 1.360 | 0.102 | 0.131 |  | 1.000 | 1.000 |
| Etobicoke | White Sucker | 8.455 | 1.432 | 0.091 | 0.075 |  | 1.000 | 1.000 |
| Highland | Blacknose Dace | -163.000 | -2.398 | 0.037 | 0.030 | * | -1.000 | 0.213 |
| Highland | Brook Stickleback | 0.400 | 1.633 | 0.089 | 0.252 |  | 1.000 | 0.756 |
| Highland | Creek Chub | 5.800 | 1.289 | 0.133 | 0.183 |  | 1.000 | 0.730 |
| Highland | Fathead Minnow | -10.200 | -1.525 | 0.101 | 0.097 |  | -1.000 | 0.486 |
| Highland | Goldfish | 2.400 | 1.000 | 0.187 | 0.509 |  | 1.000 | 0.756 |
| Highland | Longnose Dace | -28.800 | -0.875 | 0.216 | 0.319 |  | -1.000 | 0.756 |
| Highland | White Sucker | 4.000 | 2.390 | 0.038 | 0.066 |  | 1.000 | 0.394 |
| Mimico | Brook Stickleback | -0.333 | -1.000 | 0.211 | 0.498 |  | -1.000 | 1.000 |
| Mimico | Creek Chub | -23.667 | -2.086 | 0.086 | 0.121 |  | -1.000 | 0.482 |
| Mimico | Fathead Minnow | -9.000 | -0.782 | 0.258 | 0.499 |  | -1.000 | 1.000 |
| Mimico | White Sucker | 2.000 | 1.000 | 0.211 | 0.499 |  | 1.000 | 1.000 |
| Rouge | American Brook Lamprey | 0.318 | 1.578 | 0.065 | 0.133 |  | 1.000 | 1.000 |
| Rouge | Blacknose Dace | 13.773 | 1.499 | 0.074 | 0.073 |  | 1.000 | 1.000 |
| Rouge | Bluegill | -0.045 | -1.000 | 0.164 | 0.504 |  | -1.000 | 1.000 |
| Rouge | Bluntnose Minnow | -2.455 | -0.773 | 0.224 | 0.324 |  | -1.000 | 1.000 |
| Rouge | Brook Stickleback | 2.000 | 1.283 | 0.107 | 0.128 |  | 1.000 | 1.000 |
| Rouge | Brook Trout | -0.091 | -1.000 | 0.164 | 0.500 |  | -1.000 | 1.000 |
| Rouge | Brown Bullhead | -0.045 | -1.000 | 0.164 | 0.505 |  | -1.000 | 1.000 |
| Rouge | Brown Trout | 1.500 | 1.136 | 0.134 | 0.130 |  | 1.000 | 1.000 |
| Rouge | Central Stoneroller | 5.409 | 1.014 | 0.161 | 0.254 |  | 1.000 | 1.000 |
| Rouge | Common Carp | -0.091 | -0.162 | 0.436 | 0.372 |  | -1.000 | 1.000 |
| Rouge | Common Shiner | 3.409 | 1.964 | 0.031 | 0.035 | * | 1.000 | 0.983 |
| Rouge | Creek Chub | 3.000 | 0.422 | 0.339 | 0.366 |  | 1.000 | 1.000 |
| Rouge | Leuciscidae | -1.909 | -0.483 | 0.317 | 0.508 |  | -1.000 | 1.000 |
| Rouge | Fathead Minnow | -4.500 | -0.862 | 0.199 | 0.410 |  | -1.000 | 1.000 |
| Rouge | Goldfish | 0.136 | 1.368 | 0.093 | 0.247 |  | 1.000 | 1.000 |
| Rouge | Hornyhead Chub | 1.000 | 1.755 | 0.047 | 0.061 |  | 1.000 | 1.000 |
| Rouge | Johnny Darter | 5.364 | 1.333 | 0.098 | 0.116 |  | 1.000 | 1.000 |
| Rouge | Largemouth Bass | -0.182 | -1.702 | 0.052 | 0.121 |  | -1.000 | 1.000 |
| Rouge | Longnose Dace | -8.136 | -0.970 | 0.171 | 0.171 |  | -1.000 | 1.000 |
| Rouge | Mottled Sculpin | -0.727 | -1.891 | 0.036 | 0.017 | * | -1.000 | 0.521 |
| Rouge | Northern Redbelly Dace | 0.591 | 1.084 | 0.145 | 0.254 |  | 1.000 | 1.000 |
| Rouge | Pumpkinseed | 1.409 | 1.751 | 0.047 | 0.021 | * | 1.000 | 0.621 |
| Rouge | Rainbow Darter | 3.045 | 0.914 | 0.186 | 0.196 |  | 1.000 | 1.000 |
| Rouge | Rainbow Trout | 4.045 | 1.475 | 0.078 | 0.098 |  | 1.000 | 1.000 |
| Rouge | Redside Dace | 0.409 | 1.682 | 0.054 | 0.095 |  | 1.000 | 1.000 |
| Rouge | Rock Bass | 1.364 | 1.928 | 0.034 | 0.029 | * | 1.000 | 0.853 |
| Rouge | Rosyface Shiner | 0.045 | 1.000 | 0.164 | 0.503 |  | 1.000 | 1.000 |
| Rouge | Sand Shiner | 0.045 | 1.000 | 0.164 | 0.507 |  | 1.000 | 1.000 |
| Rouge | Smallmouth Bass | 0.409 | 1.000 | 0.164 | 0.211 |  | 1.000 | 1.000 |
| Rouge | Stonecat | -0.182 | -0.450 | 0.329 | 0.470 |  | -1.000 | 1.000 |
| Rouge | White Sucker | -0.864 | -0.087 | 0.466 | 0.486 |  | -1.000 | 1.000 |

Table S8: Paired *t*-test results for stream orders sorted by stream order. Strahler order 1 is not included as only one site with stream order 1 is found within the study, precluding a paired t-test approach.

| Strahler | Species | Mean  (T1-T2) | t.stat | p.param | p.perm | p<=0.05 | Sign(T1-T2) | p.holm.adj |
| --- | --- | --- | --- | --- | --- | --- | --- | --- |
| 2 | Blacknose Dace | -40.417 | -1.154 | 0.137 | 0.151 |  | -1.000 | 1.000 |
| 2 | Bluntnose Minnow | -0.167 | -1.000 | 0.169 | 0.503 |  | -1.000 | 1.000 |
| 2 | Brook Stickleback | 0.167 | 0.229 | 0.411 | 0.435 |  | 1.000 | 1.000 |
| 2 | Brown Bullhead | -0.083 | -1.000 | 0.169 | 0.504 |  | -1.000 | 1.000 |
| 2 | Brown Trout | 2.333 | 0.962 | 0.178 | 0.501 |  | 1.000 | 1.000 |
| 2 | Common Shiner | 0.083 | 1.000 | 0.169 | 0.509 |  | 1.000 | 1.000 |
| 2 | Creek Chub | -1.417 | -0.132 | 0.449 | 0.453 |  | -1.000 | 1.000 |
| 2 | Fathead Minnow | -12.750 | -1.341 | 0.104 | 0.083 |  | -1.000 | 1.000 |
| 2 | Goldfish | 1.000 | 1.000 | 0.169 | 0.506 |  | 1.000 | 1.000 |
| 2 | Johnny Darter | -0.417 | -0.270 | 0.396 | 0.500 |  | -1.000 | 1.000 |
| 2 | Longnose Dace | -23.583 | -1.841 | 0.046 | 0.012 | * | -1.000 | 0.161 |
| 2 | Mottled Sculpin | -0.250 | -1.000 | 0.169 | 0.504 |  | -1.000 | 1.000 |
| 2 | Pumpkinseed | 0.083 | 1.000 | 0.169 | 0.499 |  | 1.000 | 1.000 |
| 2 | White Sucker | 2.833 | 1.306 | 0.109 | 0.122 |  | 1.000 | 1.000 |
| 3 | American Brook Lamprey | 0.333 | 1.000 | 0.169 | 0.502 |  | 1.000 | 1.000 |
| 3 | Blacknose Dace | -45.083 | -2.165 | 0.027 | 0.013 | * | -1.000 | 0.361 |
| 3 | Bluegill | -0.083 | -1.000 | 0.169 | 0.499 |  | -1.000 | 1.000 |
| 3 | Bluntnose Minnow | 1.583 | 1.000 | 0.169 | 0.507 |  | 1.000 | 1.000 |
| 3 | Brook Stickleback | 7.833 | 1.423 | 0.091 | 0.100 |  | 1.000 | 1.000 |
| 3 | Brook Trout | -0.167 | -1.000 | 0.169 | 0.491 |  | -1.000 | 1.000 |
| 3 | Brown Bullhead | -0.417 | -1.000 | 0.169 | 0.513 |  | -1.000 | 1.000 |
| 3 | Brown Trout | 0.417 | 1.449 | 0.088 | 0.251 |  | 1.000 | 1.000 |
| 3 | Central Mudminnow | -0.083 | -1.000 | 0.169 | 0.504 |  | -1.000 | 1.000 |
| 3 | Central Stoneroller | -3.333 | -1.474 | 0.084 | 0.255 |  | -1.000 | 1.000 |
| 3 | Common Carp | -0.083 | -1.000 | 0.169 | 0.507 |  | -1.000 | 1.000 |
| 3 | Common Shiner | 0.167 | 0.132 | 0.449 | 0.498 |  | 1.000 | 1.000 |
| 3 | Creek Chub | 5.250 | 0.731 | 0.240 | 0.451 |  | 1.000 | 1.000 |
| 3 | Leuciscidae | 0.417 | 1.000 | 0.169 | 0.503 |  | 1.000 | 1.000 |
| 3 | Fathead Minnow | -4.667 | -1.532 | 0.077 | 0.044 | * | -1.000 | 1.000 |
| 3 | Goldfish | 0.167 | 1.000 | 0.169 | 0.509 |  | 1.000 | 1.000 |
| 3 | Green Sunfish | -21.250 | -1.000 | 0.169 | 0.496 |  | -1.000 | 1.000 |
| 3 | Green Sunfish x Pumpkinseed | -2.000 | -1.000 | 0.169 | 0.505 |  | -1.000 | 1.000 |
| 3 | Johnny Darter | 6.750 | 1.388 | 0.096 | 0.122 |  | 1.000 | 1.000 |
| 3 | Largemouth Bass | -0.083 | -1.000 | 0.169 | 0.499 |  | -1.000 | 1.000 |
| 3 | Longnose Dace | -8.417 | -0.775 | 0.227 | 0.233 |  | -1.000 | 1.000 |
| 3 | Mottled Sculpin | -0.083 | -1.000 | 0.169 | 0.499 |  | -1.000 | 1.000 |
| 3 | Northern Redbelly Dace | 0.083 | 1.000 | 0.169 | 0.500 |  | 1.000 | 1.000 |
| 3 | Pumpkinseed | -1.000 | -1.095 | 0.148 | 0.255 |  | -1.000 | 1.000 |
| 3 | Rainbow Darter | -2.417 | -1.000 | 0.169 | 0.499 |  | -1.000 | 1.000 |
| 3 | Rainbow Trout | 4.333 | 0.959 | 0.179 | 0.495 |  | 1.000 | 1.000 |
| 3 | Redside Dace | 0.167 | 1.000 | 0.169 | 0.507 |  | 1.000 | 1.000 |
| 3 | Smallmouth Bass | 0.333 | 0.616 | 0.275 | 0.501 |  | 1.000 | 1.000 |
| 3 | White Sucker | 9.083 | 1.807 | 0.049 | 0.007 | * | 1.000 | 0.215 |
| 4 | American Brook Lamprey | 0.538 | 1.620 | 0.066 | 0.123 |  | 1.000 | 1.000 |
| 4 | Blacknose Dace | 24.923 | 1.637 | 0.064 | 0.061 |  | 1.000 | 1.000 |
| 4 | Bluntnose Minnow | 1.231 | 1.222 | 0.123 | 0.138 |  | 1.000 | 1.000 |
| 4 | Brook Stickleback | 1.231 | 1.888 | 0.042 | 0.064 |  | 1.000 | 1.000 |
| 4 | Central Stoneroller | -0.692 | -1.128 | 0.141 | 0.245 |  | -1.000 | 1.000 |
| 4 | Common Shiner | 1.538 | 0.695 | 0.250 | 0.251 |  | 1.000 | 1.000 |
| 4 | Creek Chub | 3.692 | 0.486 | 0.318 | 0.345 |  | 1.000 | 1.000 |
| 4 | Leuciscidae | 1.769 | 1.438 | 0.088 | 0.265 |  | 1.000 | 1.000 |
| 4 | Fathead Minnow | -6.308 | -0.754 | 0.233 | 0.361 |  | -1.000 | 1.000 |
| 4 | Golden Shiner | 1.615 | 1.000 | 0.169 | 0.492 |  | 1.000 | 1.000 |
| 4 | Goldfish | 0.077 | 1.000 | 0.169 | 0.504 |  | 1.000 | 1.000 |
| 4 | Green Sunfish | -0.692 | -1.000 | 0.169 | 0.495 |  | -1.000 | 1.000 |
| 4 | Green Sunfish x Pumpkinseed | -0.077 | -1.000 | 0.169 | 0.500 |  | -1.000 | 1.000 |
| 4 | Johnny Darter | 7.308 | 1.484 | 0.082 | 0.081 |  | 1.000 | 1.000 |
| 4 | Largemouth Bass | -0.231 | -1.389 | 0.095 | 0.246 |  | -1.000 | 1.000 |
| 4 | Longnose Dace | -14.000 | -0.679 | 0.255 | 0.341 |  | -1.000 | 1.000 |
| 4 | Mottled Sculpin | -3.923 | -1.892 | 0.041 | 0.032 | * | -1.000 | 0.790 |
| 4 | Northern Hog Sucker | -0.462 | -1.000 | 0.169 | 0.502 |  | -1.000 | 1.000 |
| 4 | Northern Redbelly Dace | 0.923 | 1.000 | 0.169 | 0.508 |  | 1.000 | 1.000 |
| 4 | Pumpkinseed | 1.231 | 0.739 | 0.237 | 0.268 |  | 1.000 | 1.000 |
| 4 | Rainbow Darter | -0.462 | -0.193 | 0.425 | 0.465 |  | -1.000 | 1.000 |
| 4 | Rainbow Trout | 2.769 | 1.212 | 0.124 | 0.187 |  | 1.000 | 1.000 |
| 4 | Redside Dace | 0.538 | 1.395 | 0.094 | 0.182 |  | 1.000 | 1.000 |
| 4 | Rock Bass | 1.769 | 1.577 | 0.070 | 0.125 |  | 1.000 | 1.000 |
| 4 | Spottail Shiner | 0.846 | 1.000 | 0.169 | 0.502 |  | 1.000 | 1.000 |
| 4 | White Sucker | 16.385 | 2.483 | 0.014 | 0.004 | * | 1.000 | 0.099 |
| 5 | Blacknose Dace | -28.231 | -0.981 | 0.173 | 0.198 |  | -1.000 | 1.000 |
| 5 | Bluntnose Minnow | 3.923 | 0.637 | 0.268 | 0.265 |  | 1.000 | 1.000 |
| 5 | Brook Stickleback | 0.538 | 0.720 | 0.243 | 0.378 |  | 1.000 | 1.000 |
| 5 | Brown Bullhead | -0.308 | -1.000 | 0.169 | 0.499 |  | -1.000 | 1.000 |
| 5 | Central Stoneroller | 3.692 | 0.346 | 0.368 | 0.374 |  | 1.000 | 1.000 |
| 5 | Common Carp | -0.692 | -1.000 | 0.169 | 0.502 |  | -1.000 | 1.000 |
| 5 | Common Shiner | -15.615 | -1.549 | 0.074 | 0.062 |  | -1.000 | 1.000 |
| 5 | Creek Chub | -1.077 | -0.510 | 0.310 | 0.327 |  | -1.000 | 1.000 |
| 5 | Leuciscidae | 1.692 | 0.865 | 0.202 | 0.500 |  | 1.000 | 1.000 |
| 5 | Emerald Shiner | -0.615 | -1.000 | 0.169 | 0.498 |  | -1.000 | 1.000 |
| 5 | Fantail Darter | -1.231 | -1.000 | 0.169 | 0.496 |  | -1.000 | 1.000 |
| 5 | Fathead Minnow | 0.769 | 0.891 | 0.195 | 0.248 |  | 1.000 | 1.000 |
| 5 | Golden Shiner | 1.385 | 2.250 | 0.022 | 0.035 | * | 1.000 | 1.000 |
| 5 | Green Sunfish | -0.308 | -1.760 | 0.052 | 0.127 |  | -1.000 | 1.000 |
| 5 | Hornyhead Chub | 1.615 | 1.723 | 0.055 | 0.128 |  | 1.000 | 1.000 |
| 5 | Johnny Darter | -6.462 | -0.912 | 0.190 | 0.246 |  | -1.000 | 1.000 |
| 5 | Logperch | -0.077 | -1.000 | 0.169 | 0.502 |  | -1.000 | 1.000 |
| 5 | Longnose Dace | -7.615 | -0.256 | 0.401 | 0.409 |  | -1.000 | 1.000 |
| 5 | Mottled Sculpin | -0.077 | -1.000 | 0.169 | 0.493 |  | -1.000 | 1.000 |
| 5 | Northern Hog Sucker | 0.077 | 1.000 | 0.169 | 0.505 |  | 1.000 | 1.000 |
| 5 | Pumpkinseed | 1.692 | 1.314 | 0.107 | 0.033 | * | 1.000 | 1.000 |
| 5 | Rainbow Darter | 6.538 | 1.609 | 0.067 | 0.062 |  | 1.000 | 1.000 |
| 5 | Rainbow Trout | 0.077 | 1.000 | 0.169 | 0.500 |  | 1.000 | 1.000 |
| 5 | Rock Bass | 0.231 | 0.898 | 0.193 | 0.380 |  | 1.000 | 1.000 |
| 5 | Rosyface Shiner | 0.077 | 1.000 | 0.169 | 0.502 |  | 1.000 | 1.000 |
| 5 | Round Goby | -6.000 | -1.057 | 0.156 | 0.253 |  | -1.000 | 1.000 |
| 5 | Sand Shiner | 0.077 | 1.000 | 0.169 | 0.488 |  | 1.000 | 1.000 |
| 5 | Smallmouth Bass | 0.462 | 1.000 | 0.169 | 0.506 |  | 1.000 | 1.000 |
| 5 | Spotfin Shiner | 0.462 | 1.196 | 0.127 | 0.251 |  | 1.000 | 1.000 |
| 5 | Spottail Shiner | 0.308 | 1.298 | 0.109 | 0.257 |  | 1.000 | 1.000 |
| 5 | Stonecat | 0.231 | 0.898 | 0.193 | 0.372 |  | 1.000 | 1.000 |
| 5 | White Sucker | 3.538 | 1.630 | 0.065 | 0.069 |  | 1.000 | 1.000 |
| 6 | Blacknose Dace | -19.667 | -1.113 | 0.191 | 0.126 |  | -1.000 | 1.000 |
| 6 | Bluntnose Minnow | -25.667 | -1.314 | 0.160 | 0.251 |  | -1.000 | 1.000 |
| 6 | Common Carp | 2.667 | 1.000 | 0.211 | 0.502 |  | 1.000 | 1.000 |
| 6 | Common Shiner | 1.000 | 0.655 | 0.290 | 0.502 |  | 1.000 | 1.000 |
| 6 | Creek Chub | -3.333 | -3.780 | 0.032 | 0.128 |  | -1.000 | 1.000 |
| 6 | Leuciscidae | -27.000 | -1.000 | 0.211 | 0.494 |  | -1.000 | 1.000 |
| 6 | Fathead Minnow | 0.333 | 1.000 | 0.211 | 0.499 |  | 1.000 | 1.000 |
| 6 | Hornyhead Chub | 0.333 | 1.000 | 0.211 | 0.510 |  | 1.000 | 1.000 |
| 6 | Johnny Darter | -6.333 | -1.000 | 0.211 | 0.500 |  | -1.000 | 1.000 |
| 6 | Logperch | 0.333 | 1.000 | 0.211 | 0.493 |  | 1.000 | 1.000 |
| 6 | Longnose Dace | -11.667 | -0.796 | 0.255 | 0.376 |  | -1.000 | 1.000 |
| 6 | Pumpkinseed | -0.333 | -1.000 | 0.211 | 0.505 |  | -1.000 | 1.000 |
| 6 | Rainbow Darter | 2.333 | 0.261 | 0.409 | 0.508 |  | 1.000 | 1.000 |
| 6 | Rock Bass | 1.667 | 1.000 | 0.211 | 0.510 |  | 1.000 | 1.000 |
| 6 | Smallmouth Bass | -0.333 | -0.378 | 0.371 | 0.501 |  | -1.000 | 1.000 |
| 6 | Stonecat | -2.333 | -0.819 | 0.249 | 0.492 |  | -1.000 | 1.000 |
| 6 | White Sucker | -71.667 | -1.230 | 0.172 | 0.122 |  | -1.000 | 1.000 |

Table S9: Paired *t*-tests results for high value TBI sites.

| Species | Mean  (T1-T2) | t.stat | p.param | p.perm | p<=  0.05 | Sign  (T1-T2) | p.holm.adj |
| --- | --- | --- | --- | --- | --- | --- | --- |
| American Brook Lamprey | 0.571 | 1.000 | 0.178 | 0.502 |  | 1.000 | 1.000 |
| Blacknose Dace | -51.571 | -0.857 | 0.212 | 0.372 |  | -1.000 | 1.000 |
| Bluntnose Minnow | 11.143 | 1.324 | 0.117 | 0.248 |  | 1.000 | 1.000 |
| Brook Stickleback | 9.714 | 1.088 | 0.159 | 0.249 |  | 1.000 | 1.000 |
| Central Mudminnow | -0.143 | -1.000 | 0.178 | 0.499 |  | -1.000 | 1.000 |
| Common Shiner | 1.714 | 1.000 | 0.178 | 0.499 |  | 1.000 | 1.000 |
| Creek Chub | 19.429 | 1.251 | 0.129 | 0.150 |  | 1.000 | 1.000 |
| Leuciscidae | 0.714 | 1.000 | 0.178 | 0.490 |  | 1.000 | 1.000 |
| Emerald Shiner | -1.143 | -1.000 | 0.178 | 0.499 |  | -1.000 | 1.000 |
| Fathead Minnow | 0.857 | 1.353 | 0.112 | 0.159 |  | 1.000 | 1.000 |
| Green Sunfish | -36.571 | -1.005 | 0.177 | 0.240 |  | -1.000 | 1.000 |
| Green Sunfish x Pumpkinseed | -3.429 | -1.000 | 0.178 | 0.503 |  | -1.000 | 1.000 |
| Johnny Darter | 9.143 | 1.138 | 0.149 | 0.243 |  | 1.000 | 1.000 |
| Longnose Dace | -17.714 | -0.789 | 0.230 | 0.500 |  | -1.000 | 1.000 |
| Mottled Sculpin | -0.143 | -1.000 | 0.178 | 0.497 |  | -1.000 | 1.000 |
| Pumpkinseed | -1.571 | -1.000 | 0.178 | 0.493 |  | -1.000 | 1.000 |
| Redside Dace | 0.286 | 1.000 | 0.178 | 0.502 |  | 1.000 | 1.000 |
| Rock Bass | 0.143 | 1.000 | 0.178 | 0.495 |  | 1.000 | 1.000 |
| Round Goby | -0.571 | -1.000 | 0.178 | 0.504 |  | -1.000 | 1.000 |
| Smallmouth Bass | 0.857 | 1.000 | 0.178 | 0.503 |  | 1.000 | 1.000 |
| Spotfin Shiner | 0.143 | 1.000 | 0.178 | 0.500 |  | 1.000 | 1.000 |
| White Sucker | 13.571 | 1.721 | 0.068 | 0.031 | * | 1.000 | 0.671 |

Table S10: Paired *t*-tests results for TBI significant gain sites.

| Species | Mean  (T1-T2) | t.stat | p.param | p.perm | p<=  0.05 | Sign  (T1-T2) | p.holm.adj |
| --- | --- | --- | --- | --- | --- | --- | --- |
| Blacknose Dace | -112.000 | -1.141 | 0.168 | 0.251 |  | -1.000 | 1.000 |
| Brook Stickleback | 15.500 | 0.979 | 0.200 | 0.507 |  | 1.000 | 1.000 |
| Central Mudminnow | -0.250 | -1.000 | 0.196 | 0.506 |  | -1.000 | 1.000 |
| Creek Chub | -2.750 | -0.227 | 0.417 | 0.498 |  | -1.000 | 1.000 |
| Fathead Minnow | 2.000 | 4.899 | 0.008 | 0.064 |  | 1.000 | 0.643 |
| Green Sunfish | -63.750 | -1.000 | 0.196 | 0.501 |  | -1.000 | 1.000 |
| Green Sunfish x Pumpkinseed | -6.000 | -1.000 | 0.196 | 0.502 |  | -1.000 | 1.000 |
| Longnose Dace | -37.750 | -1.000 | 0.196 | 0.497 |  | -1.000 | 1.000 |
| Pumpkinseed | -2.750 | -1.000 | 0.196 | 0.497 |  | -1.000 | 1.000 |
| White Sucker | 4.000 | 1.633 | 0.100 | 0.251 |  | 1.000 | 1.000 |

Table S11: Paired *t*-tests results for TBI significant loss sites.

| Species | Mean  (T1-T2) | t.stat | p.param | p.perm | p<=  0.05 | Sign  (T1-T2) | p.holm.adj |
| --- | --- | --- | --- | --- | --- | --- | --- |
| Blacknose Dace | -149.333 | -1.163 | 0.182 | 0.253 |  | -1.000 | 1.000 |
| Brook Stickleback | -0.333 | -1.000 | 0.211 | 0.496 |  | -1.000 | 1.000 |
| Creek Chub | -3.667 | -0.215 | 0.425 | 0.500 |  | -1.000 | 1.000 |
| Fathead Minnow | 1.667 | 5.000 | 0.019 | 0.131 |  | 1.000 | 0.785 |
| Longnose Dace | -50.333 | -1.000 | 0.211 | 0.495 |  | -1.000 | 1.000 |
| White Sucker | 5.333 | 1.835 | 0.104 | 0.245 |  | 1.000 | 1.000 |
